# Reconstructing the genetic ancestry of the archaeological site in Amiternum: From Roman Imperial to early medieval times in Central Italy

**DOI:** 10.1016/j.isci.2026.117783

**Published:** 2026-10-08

**Authors:** Elisabetta Moroni, Vincenzo Agostini, Dario Ciabini, Giulia Colombo, Rosalinda Di Gerlando, Nicola Rambaldi Migliore, Osvaldo Zarivi, Ornella Semino, Alessandro Achilli, Alfonso Forgione, Anna Maria G. Poma, Anna Olivieri

**Affiliations:** 1 Department of Biology and Biotechnology “L. Spallanzani”, University of Pavia, via Ferrata 9, 27100 Pavia, Italy; 2 Molecular Biology and Transcriptomic Unit, IRCCS Mondino Foundation, via Mondino 2, 27100 Pavia, Italy; 3 Department of Clinical Medicine, Public Health, Life and Environmental Sciences, University of L’Aquila, piazzale Salvatore Tommasi 1, 67100 L’Aquila, Italy; 4 Department of Human Studies, University of L'Aquila, viale Nizza 14, L’Aquila, Italy

**Keywords:** ancient Italy, ancient DNA, archaeogenomics, Roman Empire, Middle Ages

## Abstract

Central-southern Italy is pivotal for reconstructing the population history of the peninsula, yet the genetic impact of the complex demographic past in the Abruzzo region remains largely unexplored. This study integrates archaeological context with ancient DNA analysis of 16 individuals excavated at the Cathedral of Santa Maria in Civitate in Amiternum. The sample comprises one adult male from the Roman Imperial period and 15 subadults mainly from the early medieval period. Genome-wide data reveal a sharp genetic discontinuity between the two epochs. The Roman individual mirrors other Italians from the same period, characterized by significant Near Eastern ancestry. In contrast, the early medieval group—genetically homogeneous—clusters with late antique and medieval populations from Central and Southern Italy, showing significant genetic influence from Central and Northern Europe. Overall, the medieval genetic structure differs from the Roman one and closely resembles that of modern Central and Southern Italians.

## Highlights

• Sharp genomic discontinuity between Roman Imperial and early medieval Abruzzo
• The medieval group is a homogeneous mix of Mediterranean and N. European ancestries
• Medieval Amiternum genomes show continuity with modern Central and Southern Italians
• Burial location rather than grave goods defines status in early medieval Amiternum

## Graphical abstract

**Figure:**
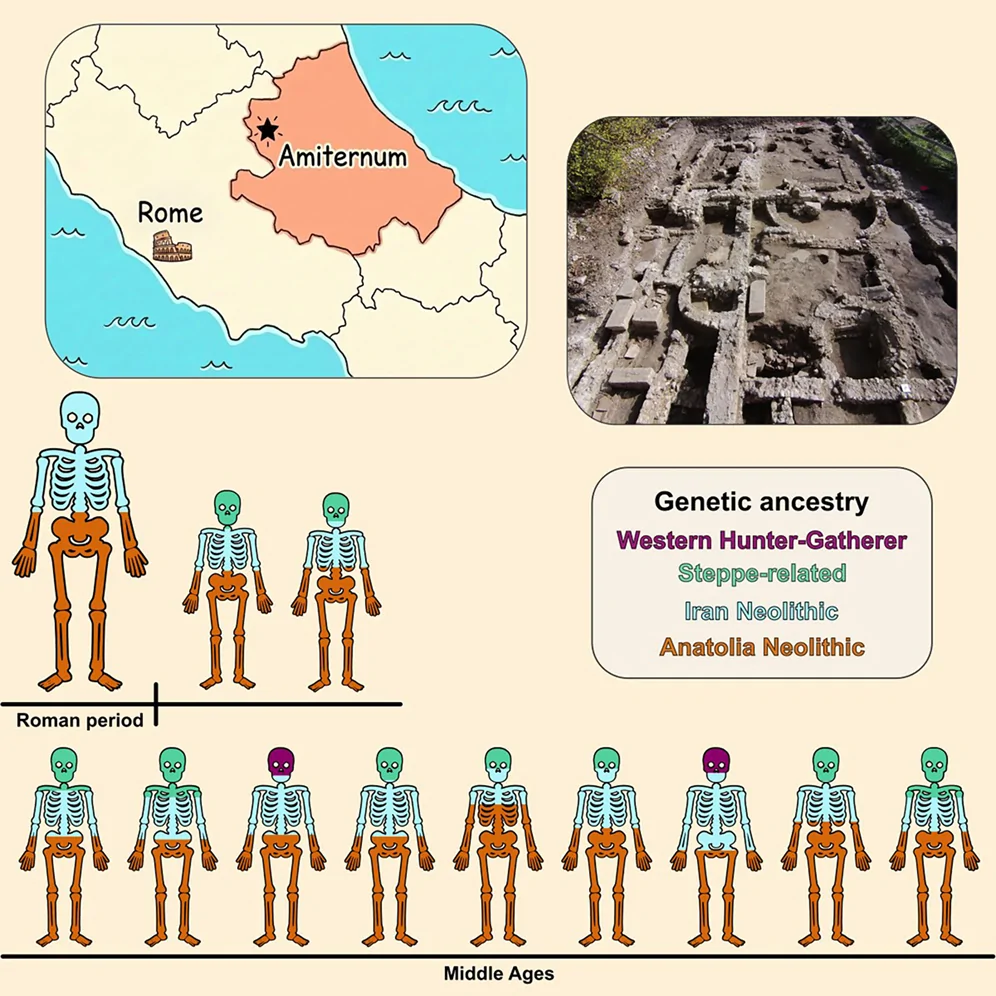

## Teaser

Paleogenetics; Archeology

## Introduction

Central-southern Italy is a key geographic region for understanding the history of the Italian peninsula, as pivotal events that shaped European history took place here. A crucial area for the conquest of Central and Southern Italy is Abruzzo, a region bordered by the Mediterranean Sea to the east, and by Marche and Latium to the north and the west. In the centuries before the Roman Empire, starting in the 7th century BCE, this area was inhabited by Italic peoples collectively known as the Samnites. These peoples occupied not only Abruzzo but also Molise and parts of Campania, Latium, and Lucania. All of them were gradually annexed by the Roman Empire, which had dominated the Abruzzo territory since the 3rd century BCE, following the Samnite Wars. In the 5th century CE, Abruzzo was among the lands occupied by Odoacer, who was defeated during the Gothic War, leading to a brief Byzantine period in the region. The arrival of the Lombards in Italy via the north-eastern frontier in 568 CE marked a crucial turning point in the redefinition of the human geography of the post-Roman peninsula. The gradual settlement of this Germanic population—more extensively documented in the Northern and Central Italian regions—had a profound impact on the political, social, and cultural structures of the conquered territories. This process resulted in the formation of the *Regnum Langobardorum*, a political entity that endured for over two centuries until the Frankish conquest of 774 CE. It also triggered significant dynamics of mobility, cultural hybridization, and territorial reorganization.^1^^,^^2^^,^^3^ The Lombards conquered Abruzzo within a few decades, probably reaching Amiternum between 580 and 595 CE via the Via Claudia-Valeria toward the Adriatic Sea.^4^ During this period, they consolidated their authority by establishing the gastaldate of Amiternum and restoring, expanding, and improving the cult buildings and burial places of the Lombard elites.^5^

Although historiography has often relegated the Abruzzo region to the margins of major Lombard and Frankish transformations, recent archaeological discoveries have revealed a more complex picture, confirming its integration into the Kingdom’s settlement and cultural dynamics. Notably, the region fell within the sphere of influence of the Duchy of Spoleto; material evidence indicates forms of occupation and territorial reorganization consistent with early medieval settlement models, which were often in continuity with Roman or Late Antique structures.^6^^,^^7^ Following the collapse of the Lombard Empire in the 8th century, Abruzzo became part of the Carolingian Frankish Empire. In the following centuries, particularly between the end of the 9th and the beginning of the 10th century, the greatest expansion of land ownership occurred.

The advent of archeogenomics has made an increasing number of ancient genomes from various epochs available, revealing many details about the genetic history of the Italian peninsula and strengthening the information provided by archaeology. To date, only a few studies have analyzed ancient genomes from the Italian Iron Age.^8^^,^^9^^,^^10^^,^^11^^,^^12^^,^^13^ During this period, Italic populations formed the cultural and genetic foundation of pre-Roman Italy, likely characterized by significant regional variation. These regional differences were defined by the multitude of peoples who crossed, conquered, and settled the Italian territory over the centuries. The Roman Empire first impacted the genetic landscape of the peninsula by introducing genetic variability through its multicultural society and introducing a strong component from the Near East.^10^^,^^14^ This component remained present even during the understudied period of Late Antiquity, which began in the 4th century CE and marked the transition from the ancient to the medieval world. The advent of the Middle Ages signaled a shift in Italian ancestry toward Central and Northern Europe, resulting in the loss of Near Eastern and Eastern Mediterranean components.^10^^,^^11^ In short, it was from the Middle Ages onwards that the inhabitants of the Italian peninsula began to resemble modern-day Italians. However, the Middle Ages did not completely flatten Italian genetic diversity; regional differences remain evident in modern Italian genomes, which still retain an exceptionally high degree of genetic variability within the European context.^15^

This study aims to integrate archaeological evidence with genomic data obtained from ancient human remains excavated in Amiternum, an ancient town in Abruzzo (Figure 1). Located a few kilometers north of L'Aquila, the current regional capital, Amiternum was founded by the Sabines around the 9th–8th century BCE and later conquered by the Romans in 290 BCE. Following the arrival of the Lombards, the town was soon abandoned due to the establishment of the nearby L'Aquila. Despite this, religious activity continued until the 13th and 14th centuries. Excavations at Amiternum have revealed the cathedral of St Mary, also known as Santa Maria in Civitate, which served as the cathedral of the diocese. Construction activity in the area where the cathedral’s archaeological remains were found dates back to public buildings of the Sabine city. These structures belong to the Roman urbanization period between the 1st century BCE and the 1st century CE, while the first documented bishops of the diocese appear in the 5th century CE. The construction of the buildings later incorporated into the early Christian cathedral began before Constantine’s Edict of 313 CE, which granted religious freedom within the Roman Empire. It is therefore likely that the original complex was subsequently expanded and transformed into a Christian cathedral in the following centuries.^16^ The cathedral remained in use until the mid-9th century, when it was downgraded to a rural parish church following its incorporation into the diocese of Rieti.

**Figure 1:**
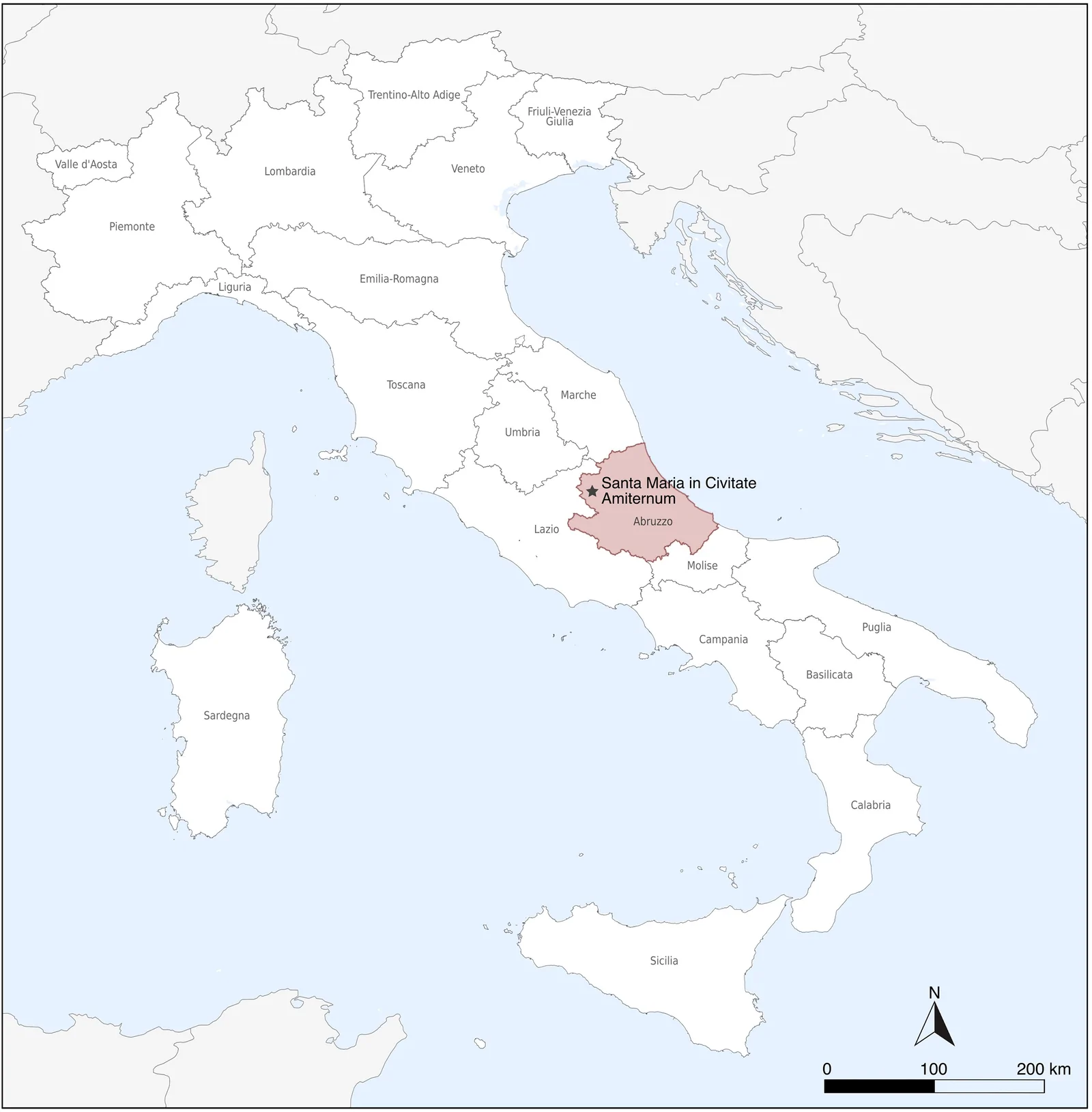
Geographical localization of the archaeological site of Santa Maria in Civitate in Amiternum (Abruzzo, Italy).

The present study analyzed 16 petrous bones from skeletal remains found in the Cathedral of Santa Maria in Civitate (referred to as ASM, standing for Amiternum Santa Maria). The samples exhibited archaeological peculiarities: one skeleton was buried outside the main body of the cathedral, in its oldest part^17^ (see the following text for further details) (Figure 2). All the other individuals were buried in the main body of the Lombard cathedral; most were located in a burial chapel, while two were found in a side chapel. The uniqueness of the archaeological site lies in the fact that the specimen buried outside the Lombard cathedral appears to be an adult male, whereas most of the other individuals buried in the cathedral (and all those selected for this genetic study) are infants or subadults.

## Results

### The archaeological context

The archaeological excavations carried out at the Cathedral of Santa Maria in Civitate in Amiternum (Abruzzo, Italy, Figure 1) allowed the collection of 16 petrous bones for genetic analyses.

Significant differences in the spatial distribution of the burials are observed (Figure 2). Thirteen individuals were interred within a funerary chapel attached to the rear wall of a structure from the late Lombard period (Building C in Forgione and Savini^18^). This structure has been archaeologically dated to between the 8th and 9th centuries. All of these burials belonged to children or infants and were devoid of grave goods, with the exception of a plain two-handled cup found in association with a double burial (S37 and S38), datable to the mid-8th century. Furthermore, one of these tombs (S23) is stratigraphically more recent and dates back to the 10th–11th century. Two additional burials were located along the northern/eastern perimeter of the church: a young woman aged approximately 17–25 years (S58), buried in a simple earthen grave, and an infant (S61), interred in the northern corner of the same building.

**Figure 2:**
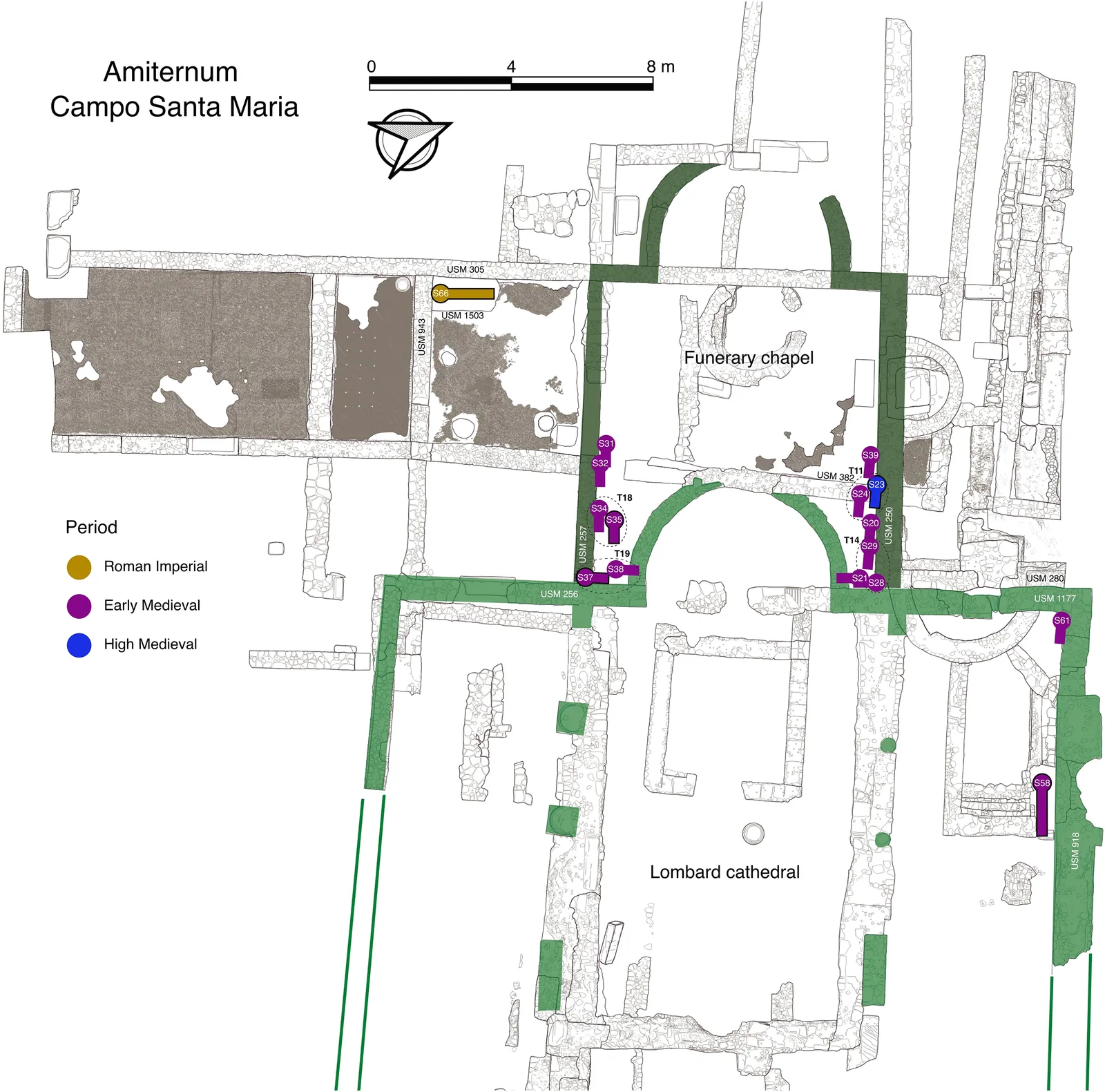
Planimetry of the archaeological site of Amiternum Santa Maria (Abruzzo, Italy) The 16 graves analyzed in this study are highlighted in dark yellow, purple, and blue according to their chronological period, as shown in the legend. Multiple burials are marked with dashed circles, with grave numbers indicated alongside (in bold), while graves containing individuals who were radiocarbon dated are outlined in black.

The entire dataset of these fifteen remains belonged to infants or young/subadult individuals (Table 1; Table S1). Seven of the remains were found in single graves (S20, S21, S31, S32, S39, S38, and S61), while the rest were found in burial chambers containing two individuals each (S23 and S24 in tomb 11, despite not being contemporaneous as mentioned earlier; S28 and S29 in tomb 14; S34 and S35 in tomb 18; and S37 and S38 in tomb 19). The specimens were too young to allow for morphological sex estimation, with the exception of S58 (Table 1; Table S1).

**Table 1:** Summary of archaeological, genetic sex estimation, radiocarbon dating, and uniparental markers for the individuals sequenced in this study

Sample ID	Sample label for analyses	Age at death	Morphological sex	Molecular sex	Archaeological dating	^14^C dating (95.4% probability)	Culture/period	Mitochondrial haplogroup (rCRS)	Y-chromosome haplogroup (ISOGG)
ASM_S20	S20	infant (<6 years)	–	M	700‑900 CE	–	early medieval	U3b2a	L1b1
ASM_S21	S21	infant (<6 years)	–	M	700‑900 CE	–	early medieval	T2b	G1a1a1b2b
ASM_S23	S23	infant (3–4 years)	–	M	800‑900 CE	1021‑1153 calCE	high medieval	K1a1b1	E1b1b1a1a1c1a
ASM_S24	S24	infant (0–2 years)	–	F	800‑900 CE	–	early medieval	K1a2	–
ASM_S28	–	infant (<6 years)	–	–	–	–	early medieval	–	–
ASM_S29	S29	infant (∼1 year)	–	F	700‑900 CE	–	early medieval	T2c1a	–
ASM_S31	S31	infant (2–3 years)	–	F	700‑900 CE	–	early medieval	U3b1	–
ASM_S32	S32	infant (∼5 years)	–	M	800‑900 CE	–	early medieval	J1b1a	J2a1a2b
ASM_S34	S34	infant (3–4 years)	–	M	700‑900 CE	–	early medieval	K1a	J2a1a1a2b2a2b2
ASM_S35	S35	infant (5–6 years)	–	M	700‑900 CE	773‑979 calCE	early medieval	K1a	J2a1a1a2b2a2b2
ASM_S37	S37	infant (<6 years)	–	M	700‑900 CE	772‑956 calCE	early medieval	K1a	J2a1a1a2b2a2b2b
ASM_S38	S38	subadult (<20 years)	–	M	700‑900 CE	–	early medieval	J1c2	J2a1a1b2a1b1b3
ASM_S39	S39	infant (1.5 years)	–	M	800‑900 CE	–	early medieval	H26a1	J2a1a1b2a1b
ASM_S58	S58	young adult (17–25 years)	F	F	800‑1000 CE	772‑978 calCE	early medieval	–	–
ASM_S61	S61	infant (<6 years)	–	F	700‑900 CE	–	early medieval	T1a1	–
ASM_S66	S66	adult (35–50 years)	M	M	300‑400 CE	241‑347 calCE	Roman Imperial	U5b2b3	R1b1a1b

The final individual analyzed, S66, was buried within a small masonry structure located outside the cathedral and not far from the aforementioned burial chamber. This chamber was dated to the 2nd to 4th centuries CE on archaeological grounds (Figure 2).^19^ S66 is also the only adult in our collection. It is estimated to be around 45–50 years old, and its morphological sex was determined to be male.

Further information on the archaeological context can be found in Data S1.

### Radiocarbon dating

Radiocarbon dates were performed on a subset of 5 out of 16 petrous bones. The dated individuals are highlighted with a thick border in Figure 2, and their dates are listed in Table 1 and illustrated in Figure S1.

All the remains excavated inside the Lombard Cathedral date back to the early medieval period, between 770 and 980 calCE. This radiocarbon dating corroborated stratigraphic analyses, as well as archaeological study of associated artifacts (Table 1; Table S1; Figure S1). For this reason, we assumed that the entire dataset collected within the Cathedral was from the same temporal period (as also outlined in Figure 2). From a historical perspective, these burials can be placed in a time frame that marks the shift from the Lombard Empire to the Frankish Carolingian conquest. The only exception is S23, which, as the stratigraphic data already indicated, appears to be the most recent, dating back to just after the year 1000.

S66, the only individual found outside the perimeter of the Lombard cathedral and the only adult male present in the burials, dates back several centuries earlier than the other samples (most of which are subadults). Radiocarbon dating places S66 in the period between 240 and 350 CE, during the Late Roman Imperial epoch. Thus, the only adult predates the Lombard period and dates back to the early centuries of construction activity on the site where the cathedral was later built.

### Genomic sequencing, molecular sex, kinship analyses, and uniparental markers

Ancient DNA (aDNA) was successfully extracted from the petrous bones of the 16 individuals and shotgun sequenced on the Illumina NextSeq550 platform (see STAR Methods for details). After excluding two individuals (namely S28 and S58) due to low genome coverage, we obtained an average depth of coverage of 0.39x and 42x for nuclear genomes and mitogenomes, respectively, with a mean endogenous DNA percentage of 49.75% (minimum 19.52%, maximum 79.76%, considering only genomes included in downstream analyses). Base misincorporation patterns were typical of aDNA and all genomes exhibited low contamination levels on both mitochondrial DNA and X chromosome (<5%) (Table S1).

Molecular sex determination was successful for 15 out of 16 individuals, revealing 10 males and 5 females (Table 1; Table S1). For S66 and S58, molecular results confirmed the osteological assessment (male and young female, respectively). The remaining 13 specimens were infants or subadults, for whom morphological sex estimation was not feasible.

Kinship analysis (Tables S1, S2, and S3) identified three individuals (S34, S35, and S37) with a first-degree relationship. S34 and S35 were buried in the same grave, while S37 was in a nearby grave, alongside the unrelated subadult S38. Two of the three first-degree related individuals (S34 and S37) were removed from downstream analyses, retaining S35 as the one with highest genome coverage. Two further samples, S31 and S32, showed a more distant degree of kinship (third-degree).

Analyses on uniparental markers corroborated the kinship findings. The three first-degree related individuals are most likely brothers, sharing the same Y-chromosome lineage (J2a1a1a2b2a2b2) and mitochondrial DNA (mtDNA) haplotype K1a (Table 1; Tables S4 and S5; Figure S2). S31 and S32, with distant autosomal relationship, differ on the maternal line (U3b1 and J1b1a, respectively), suggesting no close matrilineal connection. Nothing can be said about a possible paternal relationship, as one individual is male and the other is female.

Mitogenomes were successfully obtained from 14 individuals with virtually 100% coverage and no gaps at diagnostic positions. These mitogenomes were classified into five macro-haplogroups that are common in both modern and ancient Eurasian populations^20^: H (S39), J (S32 and S38), K (S23, S24, S34, S35, and S37), T (S21, S29, and S61), and U (S20, S31, and S66). A detailed phylogenetic tree is reported in Figure S2. Phylogenetic comparison with mitogenomes from published ancient datasets revealed that the Amiternum mitogenomes show 12 unique haplotypes that had not been identified in other ancient individuals.

Y-chromosome analysis comparing the nine ancient males with modern subjects through a phylogenetic tree^21^ identified five macro-haplogroups: E1b, J2b, L1b, G1, and R1b. E1b and J2b are commonly found in both modern and ancient populations of Western Eurasia, the Middle East, and North Africa; R1b is also frequent in modern^10^^,^^22^^,^^23^^,^^24^^,^^25^ and ancient Western Eurasian populations. The remaining two haplogroups, G1 and L1b, are rare in modern populations,^26^^,^^27^ with G1 being very rare and L1 being previously unreported among ancient individuals.

Genomic analyses were performed on the 12 unrelated genomes reaching at least 166,071 of the targeted autosomal SNPs of the 1240k SNP panel and 90,515 SNPs of Human Origins (HO) array before filtering (Table S1).

### The genomic structure of the individuals from Amiternum

We performed principal component analysis (PCA) in order to depict the genomic relationships among the 12 unrelated individuals from the archaeological site of Amiternum in the context of modern and ancient variability (Tables S6 and S7). The analysis included ancient populations from Europe and Western Asia dated back from Roman to medieval times, and the results were projected on modern variability (Figure S3).

Our results show that the medieval individuals in our dataset cluster together, with their genomic variability overlapping with that of Central and Southern Italy in both the Late Antique and medieval periods. However, the genome of the individual dating back to Imperial Rome (S66) shifts away from the genetic variability of the medieval cluster, moving closer to eastern genetic variability and overlapping with that of Roman Italy, as well as with individuals of Byzantine origin in the published ancient population dataset. This result is confirmed when ancient genomes from Amiternum are compared with modern populations: the medieval individuals mainly cluster with modern Italians from Central and Southern Italy (especially the South), while the Roman Imperial individual is closer to Middle Eastern populations (although there is no overlap) (Figure S4).

Focusing on the specific genetic variability of ancient Italy, spanning a broad time period from the Neolithic to the Middle Ages, it is clear that our individuals, both medieval and Roman, differ from the variability of Italic populations representative of the Iron Age, as has already been observed in other studies^8^^,^^13^ (Figure 3). However, the variability of present-day Italy overlaps with that of the Middle Ages. This is evident when we observe that the medieval cluster from Amiternum overlaps with modern genomes from Abruzzo and other regions of Central and Southern Italy, including Latium, Molise, Basilicata, and Apulia (Figure S5).

**Figure 3:**
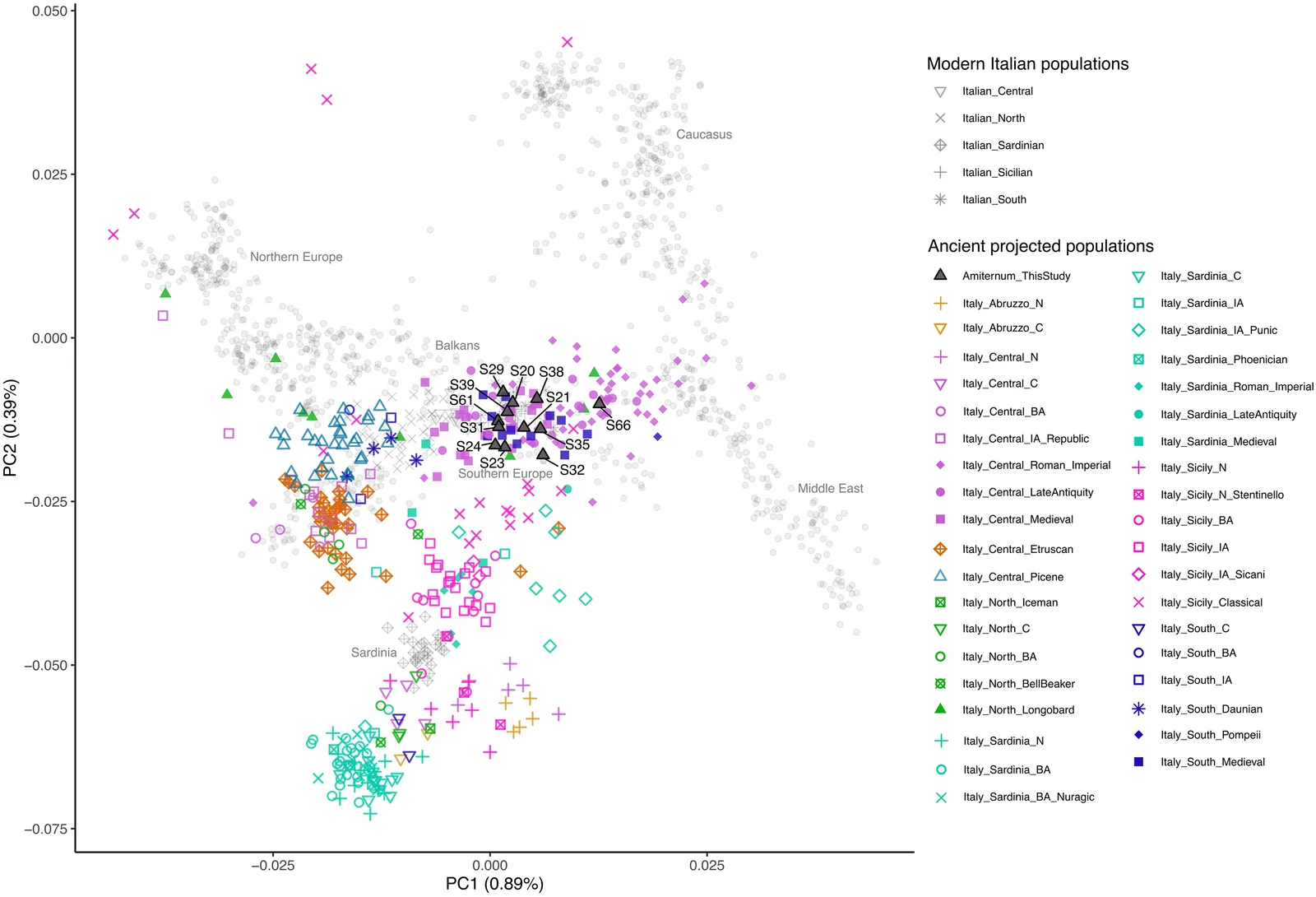
Principal component analysis plot of 385 published ancient Italian individuals dated from the Neolithic to the medieval periods (colored symbols) and the 12 ancient individuals from Amiternum (dark gray triangles) projected onto present-day genetic variability (gray dots) Modern Italians are highlighted with gray symbols. Abbreviations: N, Neolithic; C, Chalcolithic; BA, Bronze Age; IA, Iron Age.

Unsupervised ADMIXTURE analysis of modern and ancient individuals from Eurasia and North Africa (Figure 4; Figure S6) suggests a significant genetic homogeneity in the medieval samples from Amiternum, with divergence observed in the single individual from Imperial Rome (as also evidenced by the outgroup *f*3 analysis reported in Figure S7 and Table S8). Furthermore, the homogeneity of the medieval individuals contrasts starkly with the heterogeneity of ancient Italian Roman samples, even considering the time span of our dataset, which covers 300–400 years. These characteristics appear to persist over time, as evidenced by the proximity of the medieval people of Amiternum to modern Italians from Central and Southern Italy, as confirmed by both ADMIXTURE and PCA. ADMIXTURE analysis also shows a clear increase in the Western Hunter-Gatherer (WHG) component compared to the Imperial Roman individual, which has this component in minimal quantities. This component is prevalent in Italic individuals from the Iron Age and the Roman Republic but decreases until it is absent in Italians from the Roman Empire, reappearing in Late Antiquity individuals from Central Italy and becoming characteristic of medieval populations in Central and Northern Europe, such as the Lombards. A similar trend was previously observed when studying samples from Rome from different periods.^10^

**Figure 4:**
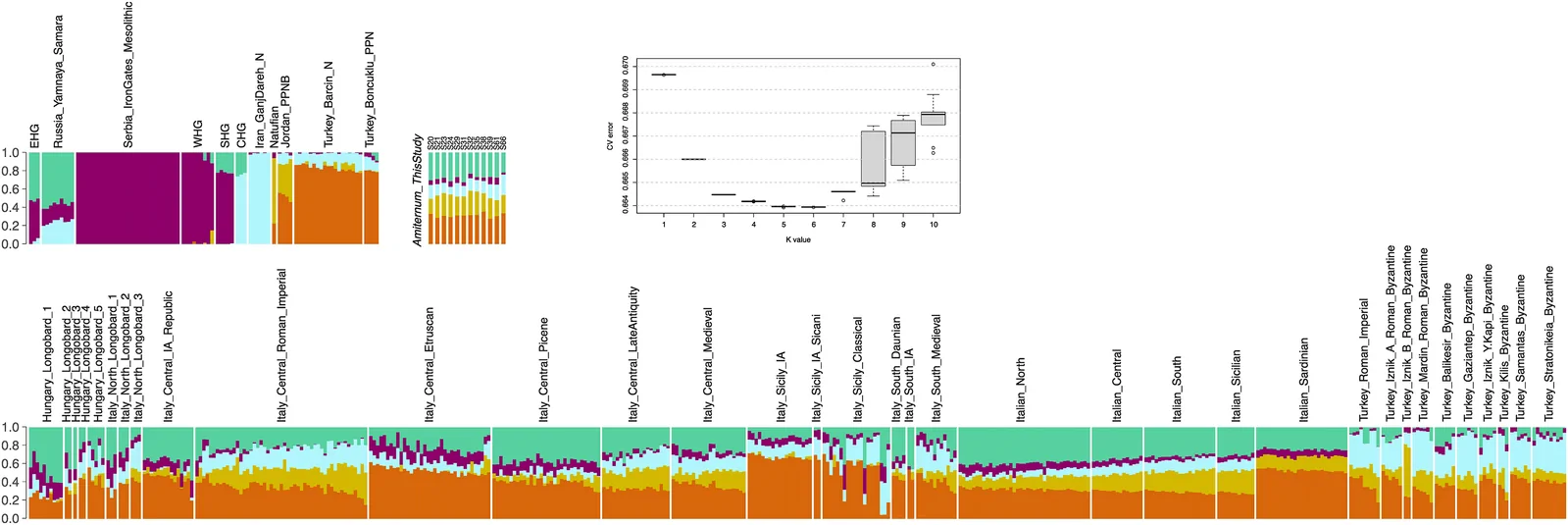
Unsupervised ADMIXTURE analysis of 1,174 modern genomes from Western Eurasia and Northern Africa merged with 1,217 published ancient individuals and the 12 individuals from Amiternum Here is shown a subset of samples at K = 5, including those representing five ancestral components that contributed to the Western Eurasian gene pool since the Bronze Age: Serbia Iron Gates Mesolithic/Western Hunter-Gatherers (WHGs)/Scandinavian Hunter-Gatherers (SHGs) (warm purple), Anatolia Neolithic (orange), Caucasian Hunter-Gatherers (CHGs)/Iran Neolithic (light blue), Eastern Hunter-Gatherers (EHGs)/Yamnaya (aqua green) and Levant Neolithic/Natufian (yellow). Amiternum individuals are indicated in italic. Inset: boxplot of 10-fold cross validation (CV) values for Ks from 1 to 10 after 10 runs.

We used qpWave to evaluate whether each pair of individuals forms a clade relative to a reference population set. In particular, this analysis allows us to clarify whether the medieval samples from Amiternum are genetically homogeneous and whether sample S66, the only individual from the Roman Imperial period in our dataset, can be considered distinct not only temporally but also genetically from the other medieval samples from Amiternum. The results (Figure S8) indicate that the medieval individuals from Amiternum form a single genetic group, meaning the population is homogeneous, as was observed in the PCA and ADMIXTURE analyses (Figures 3 and 4). Although sample S66 is not an outlier with respect to the medieval Amiternum samples, it is more cladal to the individuals from the Latium Roman Imperial population than the medieval samples are (Figure S8). Therefore, this result reinforces the interpretation that S66 and the Imperial-period individuals from Latium can be considered as a single cluster, a view further supported by their chronological contemporaneity.

As further confirmation, qpWave analysis comparing pairs of groups of individuals rather than single individuals revealed that the comparison between S66 and the Roman Imperial population from Latium does not provide a significant result (Table S9), indicating that they form a clade. In contrast, the medieval individuals from Amiternum differ significantly from both the Latium Roman Imperial group and the same group when considered together with S66 (Table S9).

To further assess the genetic differences in terms of shared drift between the medieval individuals from Amiternum and S66, who did not appear as an outlier in the qpWave analysis, we performed an *f*4 analysis comparing both groups with ancient populations from Western Eurasia and North Africa. The results (Table S11) show that the two groups are genetically distinct, primarily due to a significant excess of shared drift between the medieval Amiternum individuals and populations characterized by a WHG ancestry component, making them significantly closer to such populations.

Despite not showing a clear pattern, the outgroup *f*3 analysis in the form *f*3(Mbuti.DG; Amiternum, X), where X is an ancient population, shows that populations with greater shared drift mainly date back to ancient times (the Neolithic, Copper, and Bronze Ages) and unexpectedly not to contemporary times or periods closer to the Amiternum individuals (Figure S9 and Table S10). Furthermore, among the top 30 closest populations, there are no Italics from Central Italy (e.g., the Picenes), Italian populations from Late Antiquity, Lombard populations (Italian or from Central or Northern Europe), or Byzantines, despite their cultural and/or geographical affinity with the Amiternum individuals. The same is evident when the main differences between the Roman Imperial individual (S66) and the other medieval samples are shown together (Figure S10). In general, the amount of shared genetic drift is rather low, but the medieval Amiternum cluster shows higher levels of shared drift with external populations than the individual S66 do. However, it should be noted that, among the ancient populations related to the medieval individuals, there are several from Central and Northern Europe, albeit not from the medieval period as previously mentioned, while S66 is more closely related to ancient Mediterranean populations, including Italian ones. To explore these data further, we performed an outgroup *f*3 analysis comparing each Amiternum individual with ancient Italian populations from the Mesolithic to the Middle Ages (Figure S11; Table S10). The most recent individual in our dataset (S23 dated back to the high medieval period) was found to share the greatest genetic drift with ancient Italian populations, particularly those from ancient times, as well as more recent populations from the Late Antique and medieval periods. When ancient individuals from Amiternum are compared with modern populations (Figure S12; Table S12), it becomes evident that the individual from the Imperial Rome era differs significantly from these populations, whereas the more recent medieval individuals are much closer. This finding supports the hypothesis that significant genetic changes occurred during the Middle Ages, the effects of which can still be seen in modern populations today. Furthermore, among the top 30 modern populations with the highest shared drift, those from Central and Northern Italy (and to a lesser extent, those from Southern Italy) always appear, including the Abruzzo population in some cases.

To investigate the genetic relationships between our ancient dataset from Amiternum—separated between individuals from Imperial Rome (S66) and medieval individuals—and ancient populations from Western Eurasia and North Africa, we performed several *f*4 analyses. These analyses enabled us to verify the results of the PCA and test whether ancient Amiternum individuals from different periods were more closely related to certain populations than others. Statistically significant values were obtained when comparing our Amiternum individuals with the nearby Roman Imperial population from Latium (contemporary with S66; see Figure S13 and Table S13). Consistent with both the PCA data (Figure 3) and qpWave analyses (Figure S8), the S66 individual and the Latium samples cannot be differentiated with respect to the reference populations, whereas the medieval Amiternum ones are more closely related to Mesolithic and hunter-gatherer populations, primarily from Central and Northern Europe (Z < −3, as shown in Table S13). The same is observed when S66 and the Latium Roman Imperial individuals are grouped together (Table S13). These results also confirm the presence of an analogous component in the ADMIXTURE analysis (Figure 4) that we found to be significantly present in the medieval Amiternum individuals, but not in the one from Imperial Rome (S66). The proximity of S66 (and other Roman Imperial genomes) to Middle Eastern populations in the PCA is confirmed by statistically significant values (Z > 3) for Middle Eastern populations dating back to different eras. These populations are most similar to individuals from Latium in Imperial Rome, as also evident from similar patterns in ADMIXTURE.

The results change when individuals from Amiternum are compared with individuals from Latium dating back to the Late Antiquity (the time between the Roman Empire and the Middle Ages; see Table S14). The medieval Amiternum individuals and the one from Imperial Rome (S66) do not differ significantly from this population. Notably, when the same *f*4 is performed on S66 grouped with the Latium Roman Imperial individuals, the results become significant. The Roman Imperial group shows an excess of shared drift with Middle Eastern ancient populations (Z < −3), whereas the Late Antique population exhibits greater affinity with WHGs and WHG-related ancient groups (Z > 3, Table S14). This emphasizes that Late Antiquity most likely represents a key transitional period between the Roman Imperial and medieval eras.

However, when considering the populations of the Marche region during the Roman Imperial period (Table S15) and Late Antiquity (Table S16), S66 from Amiternum is more similar to the population of Latium than to that of the Marche, although the number of individuals available from Marche is limited compared to those from Latium—with four individuals from the Roman Imperial period and five from Late Antiquity (Table S7). Instead, the medieval individuals from Amiternum show a high affinity with the Late Antiquity ones from the Marche region. In fact, only a French Iron Age population is significantly closer to the Late Antique individuals from Marche than to those from Amiternum (Figure S14).

To quantify the contribution of different ancestry sources to our ancient individuals from Amiternum, we tested various qpAdm models on two- to four-way combinations of WHG, Anatolia Neolithic (Turkey_Barcin_N), Iran Neolithic (Iran_GanjDareh_N), Morocco_LN and Steppe-related (Russia_Yamnaya_Samara) ancestries (Table S17). Using this distal set of putative ancestral populations, we obtained working models for all individuals (Figure 5), by setting a significance threshold of *p* > 0.01 for both qpWave and qpAdm (as in previous works^13^^,^^28^), even if all individuals showed *p* values greater than 0.05, except for two individuals (S32 and S39, with 0.03 < *p* < 0.05).

**Figure 5:**
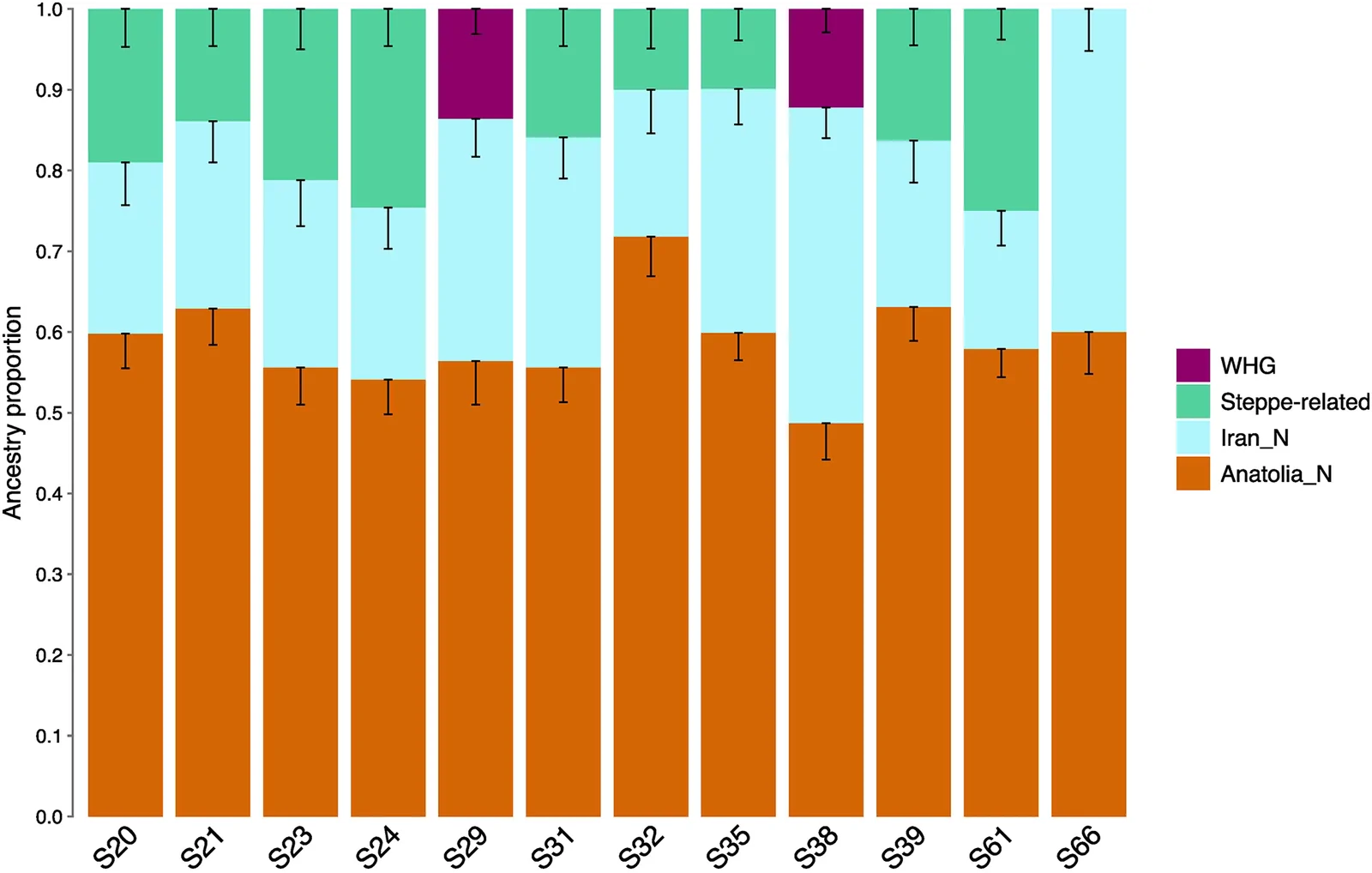
qpAdm-estimated ancestry proportions and respective standard errors for Amiternum individuals using Western Hunter-Gatherers, Anatolia Neolithic (Turkey_Barcin_N), Iran Neolithic (Iran_GanjDareh_N), and Steppe-related ancestry (Russia_Yamnaya_Samara) as distal sources Models shown represent the most parsimonious configurations identified through a model competition framework involving source rotation.

For all individuals, ancestry related to Anatolian Neolithic farmers composes the largest inferred population (ranging from 48.7% to 71.8%), with individual S32 bearing the highest value, despite not evident from ADMIXTURE analysis (Figure 4). Therefore, what differentiates the individual from one another concerns the other ancestral contributions. Notably, the individual from the Roman Imperial period (S66) is the only one whose genetic variability can be explained by just two components: Anatolian Neolithic and Iranian Neolithic, the latter of which represents 40.0%. This trend was also supported by the position of S66 in the PCA, markedly toward Near Eastern populations (Figure S3), and it was already observed in other individuals from the Roman Imperial era with whom S66 clusters (Figures S8 and S15; Tables S9, S13, and S17).^10^ The Iranian Neolithic ancestral component is also present in individuals from the medieval period; however, it never reaches the same percentage as in S66. Given that the two components present in S66, Anatolian and Iranian Neolithic, alone are not sufficient to explain their variability, the medieval individuals can be described as composed of three distal ancestries. Nine out of eleven medieval individuals exhibit Steppe-related ancestry, as represented by the Russian Yamnaya culture. By contrast, the WHG component is present in the other two medieval individuals (S29 and S38), even though they did not have the highest purple component in the unsupervised ADMIXTURE analysis.

Although Morocco_LN was included as a potential fifth source within the same qpAdm framework, none of the resulting models reached statistical significance, suggesting that this ancestral component does not significantly contribute to the genetic profile of the individuals analyzed (Table S17).

Furthermore, we tested whether the Amiternum individuals could be modeled as a mixture of Roman Imperial population from Latium and another proximal source (the left populations panel is a modified version of that used by Posth et al.^11^ for early medieval individuals from Central Italy; see STAR Methods). We found that the individual from the Roman Imperial period (S66) could be successfully modeled as deriving entirely from the Roman Imperial population from Latium. In contrast, all other individuals required a second source to achieve statistical significance, with the best-supported models featuring populations from Central or Northern Europe, such as Hungary_Longobard_1. Notably, models incorporating sources from Southern Europe or the Near East failed to reach significance, suggesting limited genetic input from these regions (Table S18). These results are consistent with a scenario in which the medieval population of Amiternum reflects admixture between local ancestry and incoming Northern European groups, likely associated with post-Roman migratory movements such as the Lombards.

## Discussion

Although Central Italy is a region of great importance in the history of the Mediterranean basin, it has received little attention from a genomic perspective. Most of the ancient genomes available from this area originate from Latium and date to the Roman period (see Figure S1C). The Abruzzo region in particular is almost entirely absent from the literature, despite its close connection to the core of ancient Roman life and its central role in cultural dynamics during later periods, especially the Middle Ages.

This study aimed to integrate archaeological and genomic data from excavations at the Cathedral of Santa Maria in Civitate in Amiternum, an ancient town near L'Aquila—the current capital of the Abruzzo region—through an interdisciplinary investigation.

The human remains analyzed in this study included sixteen petrous bones.

The archaeological context of the individuals is crucial for interpreting the genetic data and for gaining an understanding of the social dynamics of the Amiternum community during the transition from Roman Imperial to the Early Middle Ages.

The spatial distribution of the burials is particularly informative. With the exception of one individual (S66), all individuals were buried within the Santa Maria in Civitate ecclesiastical complex and are associated with funerary contexts dating from the 8th–9th centuries.^17^ Most of the sampled individuals were recovered from a funerary chapel attached to the rear wall of a building belonging to the Lombard phase of the episcopal complex. Archaeological investigations revealed a marked concentration of infant and subadult burials in this area, with at least thirteen individuals deposited in a relatively small space. Grave goods are almost entirely absent, except for a plain two-handled vessel associated with the double burial of individuals S37 and S38.

^14^C dating and genetic analyses conducted in this study confirmed that S66 dates back to around 300 CE and was an adult male. All of the other individuals were infants, subadults, or young adults. They date back at least four centuries later to around 700–900 CE (with the exception of one individual, S23, who dates back to around 1000–1100 CE) (Figure S1; Tables 1 and S1). In light of this data, we can formulate hypotheses about the archaeological context.

The concentration of juvenile burials within a privileged area directly connected to the church raises important questions regarding the social and symbolic significance of this funerary space. Similar phenomena have been documented in early medieval Italy, where burials within churches, chapels and areas closely associated with liturgical activity represented privileged access to sacred spaces. Rather than suggesting exclusion from the Christian community, the location of these burials indicates the opposite: a deliberate choice to place certain individuals in close proximity to spaces imbued with religious and commemorative significance. Identifying first-degree biological relationships among some of the individuals buried in the funerary chapel strengthens this interpretation further. The presence of related individuals (Tables S2 and S3), combined with the spatial clustering of burials and prolonged use of the same funerary area, suggests that the chapel may have functioned as a memorial space reserved for specific family groups or social units. Such practices are consistent with broader patterns documented in early medieval ecclesiastical contexts, where burials in privileged locations contributed to preserving collective memory and reinforcing family identities across generations.^29^

Archaeological evidence also suggests a gradual evolution in funerary practices. Earlier burials in the Amiternum area, particularly those outside the church walls or in peripheral locations, often included items such as bone combs, ceramic vessels, fibulae, and personal ornaments.^7^ By contrast, burials associated with the funerary chapel analyzed in this study are characterized by an almost total absence of grave goods. This may reflect broader changes in burial practices during the transition from the Lombard period to the Carolingian era, when social status and family memory were expressed more through privileged burial locations than through the deposition of material wealth in graves.

A separate discussion concerns S66, the only adult male in the dataset, dated several centuries earlier than the other individuals analyzed. While the available archaeological evidence does not permit a definitive evaluation of his social or religious status, his burial exhibits several unusual features. He was interred in a masonry funerary structure within the urban area of Amiternum at a time when such practices were still exceptional, predating the widespread adoption of Christian burial customs in urban areas. Furthermore, his grave was positioned immediately next to the corner of a building that has been preliminarily interpreted as the earliest oratory associated with the emerging Christian community of Amiternum.

Taken together, the location of the burial, its architectural setting and chronological position suggest that S66 may have held a distinctive role within the local community. As a preliminary working hypothesis, pending further archaeological evidence, it is possible to propose that this individual was a prominent member of the early Christian community of Amiternum. While this interpretation must be treated with caution, it offers a plausible explanation for the exceptional nature of the burial and its close association with what may be one of the earliest identified Christian spaces within the city.

More broadly, the funerary evidence reflects the profound transformation experienced by Amiternum between the late Roman period and the Early Middle Ages. As the monumental public spaces of the Roman city gradually lost their central role, new forms of social and religious aggregation emerged around the episcopal complex. The cemetery associated with Santa Maria in Civitate is an example of this process, demonstrating how the Christianization of space led to the formation of new centers of power, memory and community identity within the former urban fabric.

The genomic data obtained on 14 individuals enriched the archaeological data and interpretations. As already mentioned, three of these individuals were identified as first-degree relatives (Tables S2 and S3) resulting in a final dataset of 12 ancient individuals for analyses.

Although our dataset included only one individual from the Roman Imperial period, sample S66’s genetic variability mirrors that of other Roman Imperial populations in both PCA and unsupervised ADMIXTURE (Figures 3 and 4). In the PCA, S66 overlaps with Roman Imperial individuals from Latium, with whom it clusters (Figure S8; Tables S9 and S13); in the ADMIXTURE plot, it shows a reduced component associated with WHGs. As previously noted,^10^^,^^11^ the expansion of the Roman Empire (27 BCE–300 CE), which encompassed much of Europe, neighboring Asia, and the North African coasts, introduced new genetic diversity into its population. Individuals from Imperial Rome, in particular those from Latium and Marche,^10^^,^^11^^,^^12^ show strong genetic Near Eastern ancestry, a pattern clearly visible in Figure S15, where a qpAdm analysis was performed to assess ancestry proportions in ancient Italian populations for direct comparison with our dataset. The Roman Imperial period is characterized by high genetic variability and heterogeneity, as previously highlighted.^9^^,^^10^ Thirty per cent of the Roman Imperial individuals available in the literature (14 out of 45) show a genomic profile that is fully comparable with that of our Amiternum individual dated to 300 CE (S66), with only two ancestral components (Anatolian and Iranian Neolithic) capable of explaining their genetic composition. In the remaining individuals, a third Steppe-related component appears, albeit in smaller proportions. Only a few individuals (four) show the addition of a fourth WHG component, which is also associated with the North African Late Neolithic component in a single isolated genome (Figure S15; Table S17).

The genomic analyses obtained on the eleven additional individuals dated to medieval times show that, although not identical, they are fairly homogeneous. They cluster together in the PCA, exhibit similar patterns of genetic variability in the unsupervised ADMIXTURE analysis, and are cladal in qpWave heatmap (Figures 3, 4, and S8). Notably, in contrast to Iron Age Italic^8^^,^^11^^,^^13^ and Roman^10^^,^^11^^,^^30^ populations, there were no outliers in our dataset. This suggests that the Middle Ages probably saw a reduced mobility of people, and perhaps of goods, or that such movements occurred over a smaller geographical area. Therefore, although mobility across the Mediterranean basin was substantial in previous periods, probably reaching their peak during the Roman Empire,^10^ the medieval population of Amiternum appears to have been more closed. However, this may also reflect the limited sample size and the fact that the data derive from a single population from the same archaeological site.

By examining the ancestral components in nearby Italian populations dating back to the Late Antiquity and Middle Ages periods (Figure S15; Table S17), we can confirm that the medieval individuals from Amiternum reflect the same trend of change in genomic patterns and detachment from the Roman era. From Late Antiquity onwards, there has been an increase in both the Steppe-related component, present in almost all individuals, and the WHG component, which is present in 37% of individuals (seven out of 19). This latter component increased further in the medieval period, being present in 50% of individuals. Such trends are most likely due to invasions by peoples from Central and Northern Europe beginning in Late Antiquity and continuing into the medieval period, as previously noted.^10^^,^^11^ In the 6th century CE, the Germanic Lombards conquered Abruzzo, and after the collapse of the Lombard Kingdom, the region came under the rule of the Frankish Carolingian Empire, which originated in Western Europe. Our analyses reveal that the medieval population of Amiternum was subject to significant genetic influences, primarily from Central and Northern Europe, resulting in an increased presence of Steppe-related and WHG components. However, the typical Mediterranean genetic background remained. Despite the Lombard conquest, the genome of Amiternum individuals is not similar to that of Italian Lombard populations, such as those from Collegno in Northern Italy (Figure S15; Table S17). The latter (labeled Italy_North_Longobard_1 in analyses) lack the Mediterranean component linked to the Iranian Neolithic ancestry, which also characterizes Central Italians in the Roman period. Finally, the medieval individuals from Amiternum, with their mixture of ancestral components that are both typically Mediterranean and influenced by Central and Northern Europe, show continuity with modern populations from Central and Southern Italy (Figure 3; Figure S5). This reinforces the idea that the complex geopolitical history of the Italian peninsula—the birthplace of the Roman Empire and a land of passage and conquest by numerous peoples—is the main reason for the wide genetic diversity that characterizes Italian populations today compared to other European populations.^15^

### Limitations of the study

Overall, our data on ancient individuals from Central Italy suggests that the genetic structure in the medieval times differed from that in the Roman times. However, our dataset only includes one individual from the Roman Imperial period. While this individual’s genetic profile was similar to those of other Central Italians from the same period, it may not be representative of Roman Imperial populations in the Abruzzo region. Furthermore, the lack of ancient genomes from the same geographic region from both medieval and Roman times reduces the number of individuals available for direct comparison with the genetic profiles from Amiternum.

## Resource availability

### Lead contact

Further information on materials, datasets, and protocols should be directed to and will be fulfilled by the lead contact, Anna Olivieri (anna.olivieri@unipv.it).

### Materials availability

This study did not generate new unique reagents.

### Data and code availability

• FASTQ and BAM files generated in the current study have been deposited in the European Nucleotide Archive (ENA): PRJEB105378 and are publicly available as of the date of publication.
• All codes used in this study and other previously published genomic data are available at the sources referenced in the key resources table.
• Any additional information required to reanalyze the data reported in this paper is available from the lead contact upon request.

## Acknowledgments

This study received support from the Italian Ministry of University and Research (MUR) projects PRIN2022 2022BZYFBE (to O.S.), PRIN2022 2022Y8BSAL (to A.A.), and PRIN2022 20224PBY5K (to A.O.); from the 10.13039/501100006256University of L'Aquila FFO2025 FFORIC25.19 (to A.M.G.P.); and from 10.13039/501100002803Fondazione Cariplo-Bando Giovani Ricercatori 2023, rif: 2023-1373 (to N.R.M.).

## Author contributions

A.F., A.M.G.P., and A.O. conceived the study; O.Z., A.M.G.P., and A.F. collected the samples and provided archaeological background; E.M., V.A., D.C., G.C., R.D.G., and N.R.M. performed aDNA extraction and sequencing; E.M., G.C., and N.R.M. performed bioinformatic analyses; O.S., A.A., and A.O. supervised the work; E.M., G.C., N.R.M., O.Z., O.S., A.A., A.F., A.M.G.P., and A.O. discussed and interpreted the results; O.S., A.A., A.M.G.P., and A.O. acquired the funding; E.M., A.F., A.M.G.P., and A.O. wrote the original manuscript with input from all the authors. All authors read and approved the final manuscript.

## Declaration of interests

The authors declare no competing interests.

## STAR★Methods

### Key resources table

**Table:** 

REAGENT or RESOURCE	SOURCE	IDENTIFIER
**Biological samples**		

bone sample from Pars petrosa	This study	ASM_S20
bone sample from Pars petrosa	This study	ASM_S21
bone sample from Pars petrosa	This study	ASM_S23
bone sample from Pars petrosa	This study	ASM_S24
bone sample from Pars petrosa	This study	ASM_S28
bone sample from Pars petrosa	This study	ASM_S29
bone sample from Pars petrosa	This study	ASM_S31
bone sample from Pars petrosa	This study	ASM_S32
bone sample from Pars petrosa	This study	ASM_S34
bone sample from Pars petrosa	This study	ASM_S35
bone sample from Pars petrosa	This study	ASM_S37
bone sample from Pars petrosa	This study	ASM_S38
bone sample from Pars petrosa	This study	ASM_S39
bone sample from Pars petrosa	This study	ASM_S58
bone sample from Pars petrosa	This study	ASM_S61
bone sample from Pars petrosa	This study	ASM_S66

**Chemicals, peptides, and recombinant proteins**		

Isopropanol	Carlo Erba	415154
Bleach 100%	Aurogene	AU1125
Nuclease-free water	Promega	P1193
EDTA solution pH 8.0 (0.5 M) for molecular biology	PanReach Applichem ITW Reagents	A4892,0500
Proteinase K	Roche	3115852001
N-Lauryl Sarcosine Sodium Salt	Sigma-Aldrich	L9150
Qubit 1X dsDNA High Sensitivity	Thermo Fisher Scientific	Q33230
NEBNext Multiplex Oligos for Illumina (Dual Index Primers Set 2)	New England Biolabs	E7780S
TE buffer pH 7.6, RNAse free	Thermo Scientific Chemicals	J62285
Tween∗20	Carlo Erba	ABP337-500
Bovine Serum Albumine (BSA)	Promega	R3961
dNTPs 10 mM	Thermo Fisher Scientific	R0191

**Critical commercial assays**		

NEBNext® Ultra™ II DNA Library Prep Kit for Illumina	New England Biolabs	E7103L
MinElute PCR Purification Kit	Qiagen	28006
QIAquick PCR Purification Kit	Qiagen	28104
4150 TapeStation System	Agilent	G2992AA
Illumina Purification Beads	Illumina	20048236
NextSeq 500/550 v2.5 Kit	Illumina	20024904/20024908

**Deposited data**		

Shotgun data of ancient individuals	This study	ENA: PRJEB105378
Shotgun and nuclear capture data. AADR dataset v62.0	Mallick et al.^31^; Mallick et al.^32^	https://reich.hms.harvard.edu/

**Software and algorithms**		

bcl2fastq	Illumina	https://emea.support.illumina.com/sequencing/sequencing_software/bcl2fastq-conversion-software.html
SwissCal	Wacker, ETH-Zürich	N/A
OxCal v4.4	Bronk Ramsey, University of Oxford	https://c14.arch.ox.ac.uk/oxcal.html
AdapterRemoval	Schubert et al.^33^	https://github.com/mikkelschubert/adapterremoval
BWA	Li and Durbin^34^	http://bio-bwa.sourceforge.net/
MarkDuplicates	https://broadinstitute.github.io/picard/	https://broadinstitute.github.io/picard/
DeDup	Peltzer et al.^35^	https://github.com/apeltzer/DeDup
Qualimap 2	Okonechnikov et al.^36^	http://qualimap.conesalab.org/
mapDamage2.0	Jónsson et al.^37^	https://ginolhac.github.io/mapDamage/
SAMtools v.1.17	Li et al.^38^	https://www.htslib.org/doc/samtools.html
ANGSD	Korneliussen et al.^39^	https://github.com/angsd/angsd
bamUtil	Jun et al.^40^	https://genome.sph.umich.edu/wiki/BamUtil
ContamMix	Fu et al.^41^	https://github.com/plfjohnson/contamMix
Schmutzi	Renaud et al.^42^	https://github.com/grenaud/schmutzi
MAFFT	Katoh^43^; Katoh and Standley^44^	https://mafft.cbrc.jp/alignment/server/index.html
hapConX	Huang and Ringbauer^45^	https://haproh.readthedocs.io/en/latest/hapCon.html
CircularMapper	Peltzer et al.^35^	https://github.com/apeltzer/CircularMapper
HaploGrep3	Schönherr et al.^46^	https://github.com/genepi/haplogrep3
Rx	Mittnik et al.^47^	https://doi.org/10.1371/journal.pone.0163019
Ry	Skoglund et al.^48^	https://github.com/pontussk/ry_compute
pathPhynder	Martiniano et al.^21^	https://github.com/ruidlpm/pathPhynder
READv2	Alaçamlı et al.^49^	https://github.com/GuntherLab/READv2
TKGWV2	Fernandes et al.^50^	https://github.com/danimfernandes/tkgwv2
EIGENSOFT	Patterson et al.^51^	https://github.com/DReichLab/EIG
PLINK1.9	Chang et al.^52^	https://www.cog-genomics.org/plink/
ADMIXTURE	Alexander et al.^53^	https://dalexander.github.io/admixture/
ADMIXTOOLS	Patterson et al.^54^	https://github.com/DReichLab/AdmixTools
ADMIXTOOLS 2	Maier et al.^55^	https://uqrmaie1.github.io/admixtools/articles/admixtools.html

### Experimental model and study participant details

The 16 human skeletal remains analyzed in this study derive from controlled archaeological excavations conducted within the complex of Santa Maria in Civitate at Amiternum (L’Aquila, Central Italy), an ecclesiastical site characterized by long-term occupation from the Roman Imperial period through the Early Middle Ages.

The sampled individuals originate from stratigraphically defined funerary contexts associated with different structural phases of the site, including both burials located within the cathedral building and a single burial from an earlier phase outside its perimeter. All specimens were recovered under documented archaeological conditions, allowing secure association between skeletal remains and their depositional context.

The selection strategy was designed to ensure consistency between archaeological attribution and biomolecular analysis, focusing on individuals with reliable contextual information and adequate preservation for genomic investigation. The resulting dataset represents a coherent subset of the excavated population, suitable for exploring genetic patterns within a well-defined archaeological framework.

All analyses conducted on human remains were carried out only upon prior authorization from the competent Superintendency of the Italian Ministry of Culture. In particular, authorization was granted by the Superintendency for Archaeology, Fine Arts and Landscape for the Provinces of L’Aquila and Teramo (protocol number 565, 14 January 2022).

### Method details

#### ^14^C dating

^14^C dating by AMS (accelerator mass spectrometry) was performed at the CIRAM laboratories in Martillac (France) for individuals S37 and S58 and at the CEZA (Curt-Engelhorn-Zentrum Archäometrie gGmbH) laboratories in Mannheim (Germany) for individuals S23, S35 and S66. All bone samples passed quality control criteria during pretreatment, with excellent collagen yields and no signs of contamination. Resulting dates were calibrated to calendar years using the IntCal20 calibration curve^56^ and the SwissCal^57^ and OxCal v4.4^58^ software.

#### DNA extraction

16 petrous bones were available from ancient individuals for DNA extraction (Table 1) which was carried out in the dedicated clean rooms at the Department of Biology and Biotechnology of the University of Pavia, following published protocols.^59^^,^^60^ Petrous bones were soaked in sodium hypochlorite (bleach 10%) for 3 min to remove surface contamination, then washed three times with DNA-free ddH_2_O and once with isopropanol. Dried samples were placed in a DNA Crosslinker under UV. About 100 mg of bone powder from each cochlea were drilled. The powder was incubated in 1 mL of 0.5 M EDTA with 60–100 mL of Proteinase K (33.3 mg/mL) and 50 mL of 10% N-lauryl sarcosine overnight at 50°C. The DNA was purified using the MinElute Reaction Cleanup Kit (QIAGEN). The extracted DNA was quantified with Qubit 1X dsDNA HS Assay Kit.

#### Library preparation

Double-stranded DNA libraries were prepared starting from 28 μL of DNA using the NEBNext Ultra II DNA Library Prep Kit for Illumina (E7103L). After blunt-end repairing, DNA extracts were treated with USER™ enzyme for 30 min at 37°C to retain the deamination pattern only at the ends of each fragment.^61^ Adapters were used in a dilution of 1:20 and adapter-ligated libraries were purified using the MinElute Reaction Cleanup Kit (QIAGEN). Library amplification reaction was carried out as follows: 5 min at 98°C and 12 cycles of 20 s at 98°C, 15 s at 60°C, and 30 s at 72°C with a final extension at 72°C for 5 min.

Amplified libraries were purified with Illumina Purification Beads (Illumina) or with the QIAquick PCR Purification Kit (QIAGEN). Each library was quantified with Qubit 1X dsDNA HS Assay Kit and analyzed with the Agilent 4150 TapeStation system to check size distribution.

#### NGS sequencing

Shotgun sequencing was performed on the Illumina platform (Illumina NextSeq550 System) to generate low-coverage genomes. The sequencing instrument was provided by the Molecular Biology and Transcriptomic Unit at the IRCCS Mondino Foundation in Pavia (Italy).

Demultiplexing was performed using the Illumina bcl2fastq Conversion Software v2.19.0.316.^62^

We successfully sequenced 15 out of 16 DNA samples analyzed: only individual S28 had an endogenous DNA content <1%. Eventually, 14 out of 16 genomes that reached a minimum nuclear depth of 0.1x were retained for downstream analyses.

### Quantification and statistical analysis

#### Mapping

Illumina adapters were removed from raw data using AdapterRemoval v.2.3.2,^33^ which was also used to trim FASTQ files, keeping only reads longer than 30 bases after trimming and, for paired-end sequencing, collapsing reads overlapping for at least 11 bases (parameters used: --trimns –maxns 2 –trimqualities –minquality 2 –minlength 30 –collapse –minalignmentlength 11). Trimmed reads were mapped against the human reference genome hs37d5 and the mitochondrial revised Cambridge Reference Sequence (rCRS)^35^^,^^63^ in separate runs, using the algorithm aln/samse of Burrows-Wheeler Aligner (BWA) v0.7.17-r1188,^34^ with parameters tuned for ancient DNA: -l 1024 -n 0.01 -o 2. Duplicates resulting from library amplification were removed with MarkDuplicates v3.0.0^64^ for single-end sequencing runs, and DeDup v0.12.8^65^ for paired-end runs.

We assessed sequencing quality metrics, such as depth and coverage, using Qualimap v2.2.2-dev.^36^

#### aDNA authentication and softclipping

Ancient DNA damage patterns and fragment length distribution were analyzed with mapDamage 2.1.0 to prove the authenticity of the ancient DNA sequences.^37^ Eventually, the BAM files were further filtered and processed with SAMtools v1.17^38^ to keep only the reads with a minimum mapping quality of 20.

Error rate was estimated using ANGSD 0.940-dirty^39^ by comparing the excess of derived alleles in our ancient individuals to those in a high-quality, error-free individual (NA12778 from the 1000 Genomes Project, as in^66^), using the chimpanzee genome (mapped to hg19 and provided within the ANGSD package) as an outgroup to determine the ancestral allele. We used the -doAncError 1 option and ran the estError.R script provided with the ANGSD package.

Residual DNA damage was removed with bamUtil v1.0.15^40^ command trimBam, using the flag -c and with parameters -R 8 -L 2, since library amplification after adapter ligation was performed using the proofreading NEBNext Ultra II Q5 Master polymerase, causing a reduction of the deamination frequency on the 5′ end.^67^

#### Contamination estimates

Contamination rates on mitochondrial DNA (mtDNA) were estimated with ContamMix and Schmutzi, considering only results based on a minimum average mtDNA depth of 10x.

For ContamMix,^41^ we first built a consensus sequence using ANGSD v0.940-dirty^39^ with the following parameters: -minMapQ 30, -minQ 20, -doCounts 1, -setMinDepth 3, and -doFasta 2 (majority rule). Reads uniquely aligned to the mitochondrial reference sequence were then remapped to the consensus sequence using SAMtools v1.17 (fastq)^38^ and BWA v0.7.17-r1188 (aln/samse),^34^ applying the same parameters optimized for ancient DNA.

The consensus sequence was aligned to a panel of 311 worldwide mtDNA sequences,^68^ representing potential contaminants, using MAFFT v7.520.^43^^,^^44^

We ran ContamMix v1.0-10 with three independent MCMC chains of 50,000 iterations each, trimming 2 bases from the ends of reads to reduce the impact of aDNA damage. Convergence was assessed by monitoring the Gelman-Rubin diagnostic.^69^

Schmutzi^42^ was used to estimate mtDNA contamination by leveraging both deamination patterns and fragment length distributions. Present-day human contamination was evaluated through an iterative likelihood approach, using a non-redundant database of 197 human mitogenomes included in the package.

Specifically, contDeam.pl was run with the options --lengthDeam 2 --library double, and schmutzi.pl was executed with the parameters --lengthDeam 2 --uselength.

Nuclear contamination in male individuals was estimated on the X chromosome using hapConX.^45^ A pileup of the BAM files was generated using SAMtools mpileup^38^ with parameters -q 30 -Q 30 -r X and using the provided bed file derived from the 1000 Genome Project sequence data. hapConX^45^ was run with default parameters.

Samples with contamination rates lower than the 5% were retained for downstream analyses.

#### Mitochondrial DNA analysis

mtDNA reads were extracted by mapping the raw data to rCRS. After quality trimming and adapter removal, processed reads were aligned to a modified and elongated version of rCRS using the algorithm aln/samse of BWA v0.7.17-r1188^34^ and then realigned with CircularMapper v1.0^65^ specific for circular genomes. Then, duplicate reads were removed with MarkDuplicates v3.0.0^64^ for single-end sequencing runs, and DeDup v0.12.8^65^ for paired-end runs. The BAM files were further filtered and processed with SAMtools v1.17^38^ to keep only the reads with a minimum mapping quality of 20.

Consensus calling was performed with ANGSD v0.940-dirty^39^ using the parameters -minMapQ 30 -minQ 20 -doCounts 1 -setMinDepth 5 and -doFasta 2 (majority rule).

Haplogroup classification was performed with HaploGrep3 (Phylotree 17 Forensic Update v1.2).^46^ The result was validated through manual inspection of the output.

#### Sex determination and Y-chromosome haplogroup assignment

Biological sex was estimated by applying Rx^47^ and Ry^48^ methods, which rely on the ratio of sequencing reads aligning to the X chromosome and the autosomes (Rx), and to the X and Y chromosomes (Ry), respectively.

To classify the Y-chromosome haplogroup of the ten males from Amiternum, we extracted reads mapping to the Y chromosome from the merged BAM file using SAMtools v1.17.^38^ Haplogroup assignment was then performed with pathPhynder v1.2.3^21^ using default parameters. The result was validated through manual inspection of the output.

#### Kinship estimation

Relatedness between the 14 individuals from Amiternum who were eventually retained for downstream analyses was assessed using two different methods, READv2^49^ and TKGWV2,^50^ assuming pseudohaploid data.

In both cases, we used ANGSD v0.940-dirty^39^ -doHaploCall 1 to sample a random base for each position of the 1240k SNP set.

READv2^49^ allows to infer relatedness up to third degree by estimating the pairwise mismatch rate (P0) between individuals. To scale the classification cutoffs for the expected genetic distances between two unrelated individuals from the same population and using the same SNP panel, READv2^49^ performs a normalization step. This normalization is based on an estimate of the pairwise mismatch rate among unrelated individuals, which typically requires a minimum sample size of four. Therefore, we ran READv2^49^ with default settings (median) on the dataset including the 14 individuals from Amiternum.

TKGWV2^50^ is a pipeline to estimate biological relatedness up to second degree that leverages allele sharing across genome-wide variants and allele frequencies to infer the relationship between pairs of individuals. We ran TKGWV2^50^ using the population allele frequencies of modern European CEU from the 1000 Genomes Project Phase 3, as provided with the software.

Downstream analyses were restricted to 12 unrelated individuals (≥ third degree).

#### Comparative datasets for genome-wide analysis on autosomes

We compared the pseudohaploid genomes from Amiternum, generated using ANGSD v0.940-dirty^39^ -doHaploCall 1 option, with previously published modern and ancient genomes reported in the dataset of David Reich’s laboratory (AADR v62.0, last access: January 07, 2025).^31^^,^^32^ Additionally, we included 39 ancient individuals from Central Italy from^13^ to 129 modern Italians from.^15^

We selected only ancient and modern individuals that showed no issues in their group assignment or quality (i.e., those labeled “PASS”). Moreover, duplicates and possibly related individuals were excluded, as well as those with fewer than 30,000 SNPs from the 1240K + HO SNP set.

We further refined the dataset by retaining only those genomes from geographical regions relevant to this study: Western Eurasia and Northern Africa, for a total of 3157 ancient and 1323 modern individuals (Tables S6 and S7).

To maximize the number of SNPs available for each analysis we built two datasets: A) including 1240K + HO autosomal data (579,720 SNPs), for those analyses involving modern individuals (PCA, ADMIXTURE); B) including 1240k autosomal data (1,150,639 SNPs), for those analyses involving ancient individuals only (kinship, outgroup *f*3).

We used convertf from the EIGENSOFT package v7.2.0^51^ to convert the datasets into PLINK binary format. All data were subsequently merged using PLINK v1.90.^52^

In the 1240K + HO dataset, modern individuals were filtered by removing all variants with missing call rates exceeding 2% (--geno 0.02) as well as with more than 2% missing genotypes (--mind 0.02).

Furthermore, monomorphic SNPs were removed from both modern and ancient populations using a minor allele frequency threshold of 0.01 (--maf 0.01).

For the PCA analysis, SNPs with a minimum allele frequency below 5% in modern populations were also excluded (--maf 0.05).

The ancient dataset was merged with the modern one and then filtered using the --geno and --mind options set to 0.60 and 0.95, respectively, retaining only individuals with more than 10,000 SNPs after filtering.

The label for the population assigned to each individual used in this study can be found in Tables S6 and S7.

The following abbreviations were used: WHG: Western Hunter-Gatherer, EHG: Eastern Hunter-Gatherer, CHG: Caucasian Hunter-Gatherer, SHG: Scandinavian Hunter-Gatherer, N: Neolithic, PPN: Pre-Pottery Neolithic, LBK: Linearbandkeramik, C: Chalcolithic, BA: Bronze Age, IA: Iron Age, Anc: Ancient, E: Early, M: Middle, L: Late.

#### Principal component analysis (PCA) on allele frequencies

Principal component analysis (PCA) was performed using smartpca implemented in EIGENSOFT package v7.2.031,^51^ for a total of 1174 modern and 931 ancient individuals from 1240K + HO dataset. The 12 Amiternum individuals, as well as other published ancient individuals and the 129 modern Italians from^15^ (genotyped on Infinium Omni2.5-8 Illumina beadchip, thus with only few positions in common with HO and 1240k ones), were projected onto the principal components computed from 1174 present-day genomes from the 1240K + HO dataset (Tables S6 and S7), using the lsqproject and autoshrink options. PCA results were visualized using R v4.2.3.^70^

#### Clustering analysis with ADMIXTURE

Clustering analysis was performed on the 12 Amiternum individuals, merged with 1217 ancient and 1174 modern genomes, with ADMIXTURE v1.3.0^53^ after pruning the comparative dataset for linkage-disequilibrium (--indep-pairwise 200 25 0.4), thus retaining 211,602 SNPs. We performed ten independent unsupervised ADMIXTURE runs for each K, from K1 to K10, adding the --cv = 10 flag to identify the 10-fold cross-validation (CV) error for each K. The cross-validation values for each K were visualized with R v4.2.3^70^ to select the model with highest likelihood, as well as ADMIXTURE barplot.

#### Outgroup *f*3 statistics

We performed outgroup *f*3 analyses in the form *f*3(Mbuti; pop2, pop3) to investigate genetic affinities as measured by shared genetic drift between the 12 Amiternum individuals and a set of ancient and modern populations from 1240k and 1240K + HO datasets (Tables S6 and S7). Outgroup *f*3 statistics were computed with the qp3pop function implemented in ADMIXTOOLS v2.0.6^55^ with the allsnps option set to YES. Ten modern individuals of the Mbuti population were used as an outgroup.

Ancient published individuals with the same cultural/archaeological affiliation were grouped together and labels can be found in Table S7.

#### *f*4 statistics

We performed a series of *f*4 statistics in the form *f*4(Mbuti.DG, X; pop3, pop4) to investigate the genetic relationships between Amiternum individual S66, dated back to Roman Imperial period, Amiternum Early-HighMedieval cluster and a set of ancient populations (X) from the 1240k dataset (Table S7). The *f*4 was computed with the *f*4 function implemented in ADMIXTOOLS v2.0.6^55^ with the allsnps option set to YES, and ten modern individuals of the Mbuti population were used as an outgroup.

The sign of the resultant *Z* score, considered significant only if |Z|≥3, indicates the direction of the gene flow: if it is positive, the gene flow occurred between X and pop4, whereas if it is negative the gene flow occurred between X and pop3.

Ancient published individuals with the same cultural/archaeological affiliation were grouped together and labels can be found in Table S7.

#### Cladality testing with qpWave

We ran qpWave v1570 from ADMIXTOOLS v8.0.2^54^ to test cladality among the 12 individuals from Amiternum (11 medieval individuals and the Roman individual S66) and 45 previously published Roman Imperial individuals from Latium (Italy_Latium_Roman_Imperial), following the same approach as in^71^ and.^72^ All analyses were run with the options “allsnps = YES” (to maximize the number of SNPs used in each test) and “inbreed = NO” (due to the presence of populations represented by single haploid individuals). A set of 14 outgroups was employed as “right populations”: Mbuti.DG (*n* = 10), CHG (*n* = 3), EHG (*n* = 3), ElMiron (*n* = 1), Jordan_PPNB (*n* = 4), MA1 (*n* = 1), Natufian (*n* = 2), Ust_Ishim (*n* = 1), Vestonice16 (*n* = 1), WHG (*n* = 11), Turkey_Barcin_N (*n* = 19), Iran_GanjDareh_N (*n* = 6), Russia_Yamnaya_Samara (*n* = 9), and Morocco_LN (*n* = 2), with Mbuti.DG always placed in the first position. We performed qpWave tests on single individuals as well as on archaeologically defined groups (Amiternum_Early-HighMedieval, S66 and Italy_Latium_Roman_Imperial).

qpWave models with *p*-values <0.01 were considered significantly rejected, whereas pairs of individuals that passed the qpWave test at the *p*-value ≥0.01 threshold and dated to the same period were grouped for analysis. qpWave results were visualized with R v4.2.3.^70^ According to these results, S66 forms a clade with the Roman Imperial individuals from Latium and was therefore grouped with them. One Latium Roman Imperial individual (R37_R11115_merge.SG) was identified as an outlier and consequently excluded from downstream analyses (Table S9).

#### Ancestry deconvolution with qpWave/qpAdm

qpAdm analysis was performed with ADMIXTOOLS v8.0.2^54^^,^^73^ with the options “allsnps = YES” (to maximize the number of SNPs used in each test) and “inbreed = NO” (due to the presence of populations represented by single haploid individuals), to investigate the ancestral genetic composition of the 12 Amiternum individuals in greater detail.

The method does not require prior knowledge of population phylogeny; rather, it assumes that the target population is genetically related to the outgroups through a set of source populations, and that these sources exhibit asymmetric relationships with the outgroups. Under these conditions, the target can be modeled as a mixture of the source populations, with admixture proportions estimated by solving a matrix of *f*4 statistics.^74^

A set of nine outgroups was used as “right populations”: Mbuti.DG (*n* = 10), CHG (*n* = 3), EHG (*n* = 3), ElMiron (*n* = 1), Jordan_PPNB (*n* = 4), MA1 (*n* = 1), Natufian (*n* = 2), UstIshim (*n* = 1) and Vestonice16 (*n* = 1), with Mbuti.DG always in the first position.

For the distal qpAdm model we used the following five sources as “left populations”: WHG (*n* = 11), Turkey_Barcin_N (*n* = 19), Iran_GanjDareh_N (*n* = 6), Russia_Yamnaya_Samara (*n* = 9) and Morocco_LN (*n* = 2).

We first ran qpWave v1570 to assess whether the source populations were distinguishable from one another given the outgroup set, and to test if the targets could be described as a combination of these sources. For models with *p*-value ≥0.01, we used qpAdm v2050 by using a rotating approach as described in^75^ and recommended in^73^ and,^76^ with strict temporal stratification of sources and targets. We tested qpAdm models involving two to four sources, and only the most parsimonious ones with *p*-value ≥0.01 were retained for interpretation (as in^13^^,^^28^).

A two-way qpAdm analysis using a set of proximal sources was also performed following the base model approach. In this case, a set of 15 outgroups was used as “right populations”: Mbuti.DG (*n* = 10), CHG (*n* = 3), EHG (*n* = 3), ElMiron (*n* = 1), Jordan_PPNB (*n* = 4), MA1 (*n* = 1), Natufian (*n* = 2), UstIshim (*n* = 1) and Vestonice16 (*n* = 1), WHG (*n* = 11), Turkey_Barcin_N (*n* = 19), Iran_GanjDareh_N (*n* = 6), Russia_Yamnaya_Samara (*n* = 9), Morocco_LN (*n* = 2) and Italy_Central_BA (*n* = 5), with Mbuti.DG always in the first position. The 24 sources used in the proximal model as “left populations” are a modified version of those used by^11^ for early medieval genomes from Central Italy (C.Italy_Early.Medieval), with Italy_Latium_Roman_Imperial included as fixed source (Tables S7 andS18). The significance thresholds of qpWave/qpAdm are consistent with those specified for the distal model.

All feasible qpAdm models were cross-validated against PCA and unsupervised ADMIXTURE results to retain only those that are biologically plausible and to reduce false-discovery rates.^76^

## Supplemental information

Document S1. Figures S1–S15 and Data S1

Table S1. Summary of archaeological, mapping, uniparental markers, genetic sex estimation, contamination, and damage statistics for individuals sequenced in this study

Table S2. Kinship estimation performed with READ v.2

Table S3. Kinship estimation performed with TKGWV2

Table S4. mtDNA haplogroup prediction performed with Haplogrep3

Table S5. Y-Chromosome haplogroup prediction performed with pathPhynder

Table S6. Modern published individuals used for autosomal analyses

Table S7. Ancient published individuals used for autosomal analyses

Table S8. Results of outgroup *f*3 analysis on Amiternum individuals

Table S9. Results of qpWave cladality tests

Table S10. Results of outgroup *f*3 analysis with ancient comparative samples

Table S11. Results of *f*4 analyses in the form *f*4(Mbuti.DG, X; ASM_Early-HighMedieval, S66)

Table S12. Results of outgroup *f*3 analysis with modern samples from AADR and Abruzzo individuals from Raveane et al.

Table S13. Results of *f*4 analyses in the form *f*4(Mbuti.DG, X; ASM_Early-HighMedieval, Italy_Latium_Roman_Imperial), *f*4(Mbuti.DG, X; S66, Italy_Latium_Roman_Imperial), and *f*4(Mbuti.DG, X; ASM_Early-HighMedieval, Italy_Latium_Roman_Imperial_S66)

Table S14. Results of *f*4 analyses in the form *f*4(Mbuti.DG, X; ASM_Early-HighMedieval, Italy_Latium_LateAntiquity), *f*4(Mbuti.DG, X; S66, Italy_Latium_LateAntiquity), and *f*4(Mbuti.DG, X; Italy_Latium_Roman_Imperial_S66, Italy_Latium_LateAntiquity)

Table S15. Results of *f*4 analyses in the form *f*4(Mbuti.DG, X; ASM_Early-HighMedieval, Italy_Marche_Roman_Imperial), *f*4(Mbuti.DG, X; S66, Italy_Marche_Roman_Imperial), and *f*4(Mbuti.DG, X; Italy_Latium_Roman_Imperial_S66, Italy_Marche_Roman_Imperial)

Table S16. Results of *f*4 analyses in the form *f*4(Mbuti.DG, X; ASM_Early-HighMedieval, Italy_Marche_LateAntiquity), *f*4(Mbuti.DG, X; S66, Italy_Marche_LateAntiquity), and *f*4(Mbuti.DG, X; Italy_Latium_Roman_Imperial_S66, Italy_Marche_LateAntiquity)

Table S17. Results of qpAdm analysis with distal sources

Table S18. Results of qpAdm analysis with proximal sources
