# Supplementary material for: Reconstructing the genetic ancestry of the archaeological site in Amiternum: From Roman Imperial to early medieval times in Central Italy
Source: iScience. 2026 Oct 8;29(11):117783. doi: 10.1016/j.isci.2026.117783 (PMC13677311; doi:10.1016/j.isci.2026.117783)
Supplement: Supplementary file 1 [file mmc1.pdf]

iScience, Volume *29*

Supplemental information

Reconstructing the genetic ancestry of the archaeological site in Amiternum: From Roman Imperial to early medieval times in Central Italy

Elisabetta Moroni, Vincenzo Agostini, Dario Ciabini, Giulia Colombo, Rosalinda Di Gerlando, Nicola Rambaldi Migliore, Osvaldo Zarivi, Ornella Semino, Alessandro Achilli, Alfonso Forgione, Anna Maria G. Poma, and Anna Olivieri

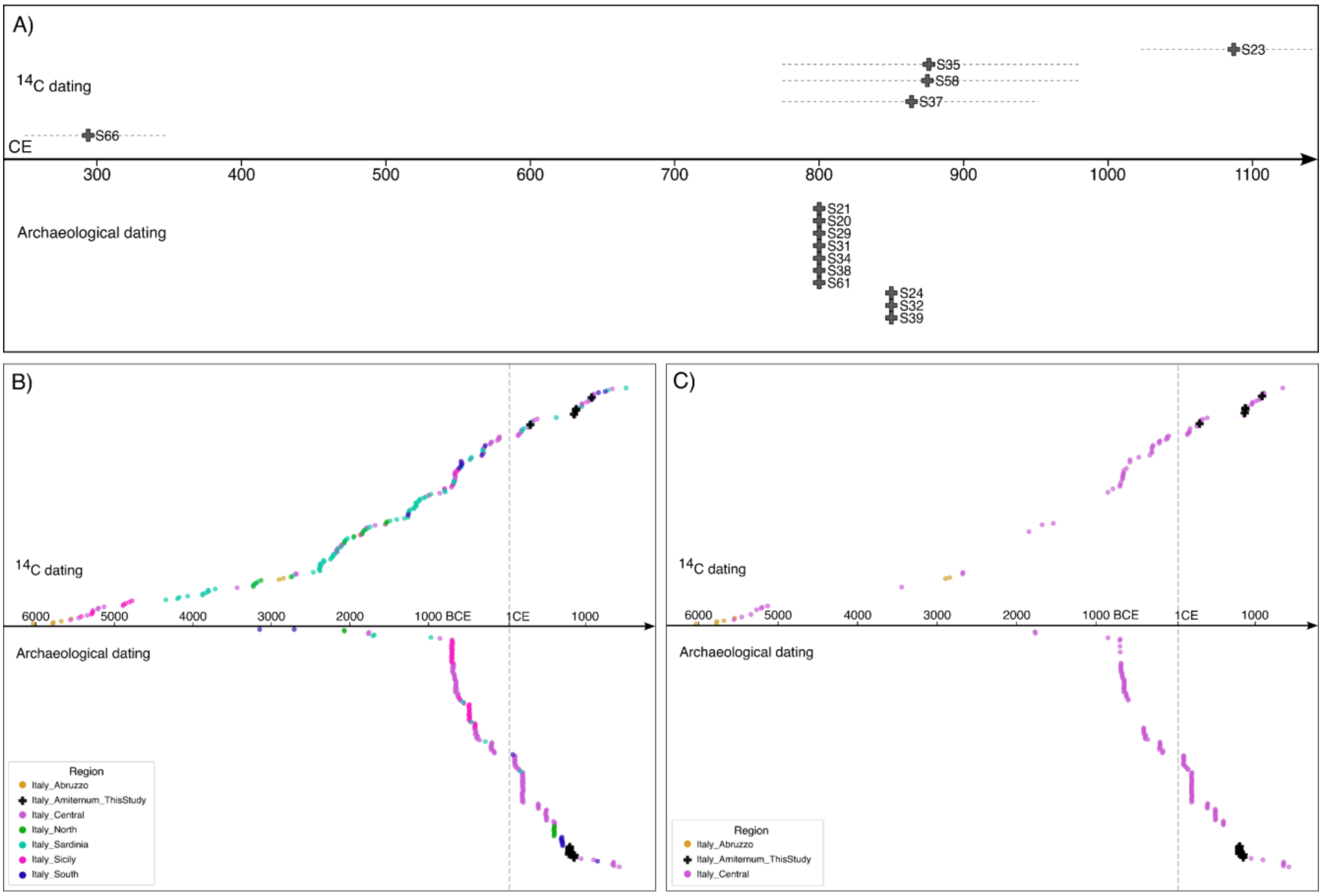

Figure S1. Chronological distribution of ancient individuals from Amiternum and other Italian regions. A) Chronological distribution (mean dates) of the Amiternum individuals analysed in this study (Italy_Amiternum_ThisStudy), spanning from ~250 CE to ~1100 CE. Samples are separated by dating method: radiocarbon-dated individuals (^{14}C dating, upper panel) and archaeologically dated individuals (lower panel). ^{14}C results were calibrated to calendar years using the IntCal20 calibration curve (STAR Methods); the mean calibrated date for each sample was calculated as the midpoint of the 95.4% (2σ) calibrated age range (Tables 1 and S1), with the full 2σ range indicated by horizontal dashed lines; B) Chronological distribution (mean dates) of Italian ancient samples dated by radiocarbon or archaeological context across Italian regions. Full dataset including samples from all Italian regions, corresponding to the individuals employed in Figure 3; C) Subset focusing on Central Italy and Abruzzo. Each point represents an individual sample, colored by region, while black crosses indicate samples from this study. The horizontal axis shows calibrated time (from ~6000 BCE to ~1200 CE), with the vertical arrangement separating radiocarbon dates (above) from archaeological attributions (below). The dashed vertical line marks 1 CE.

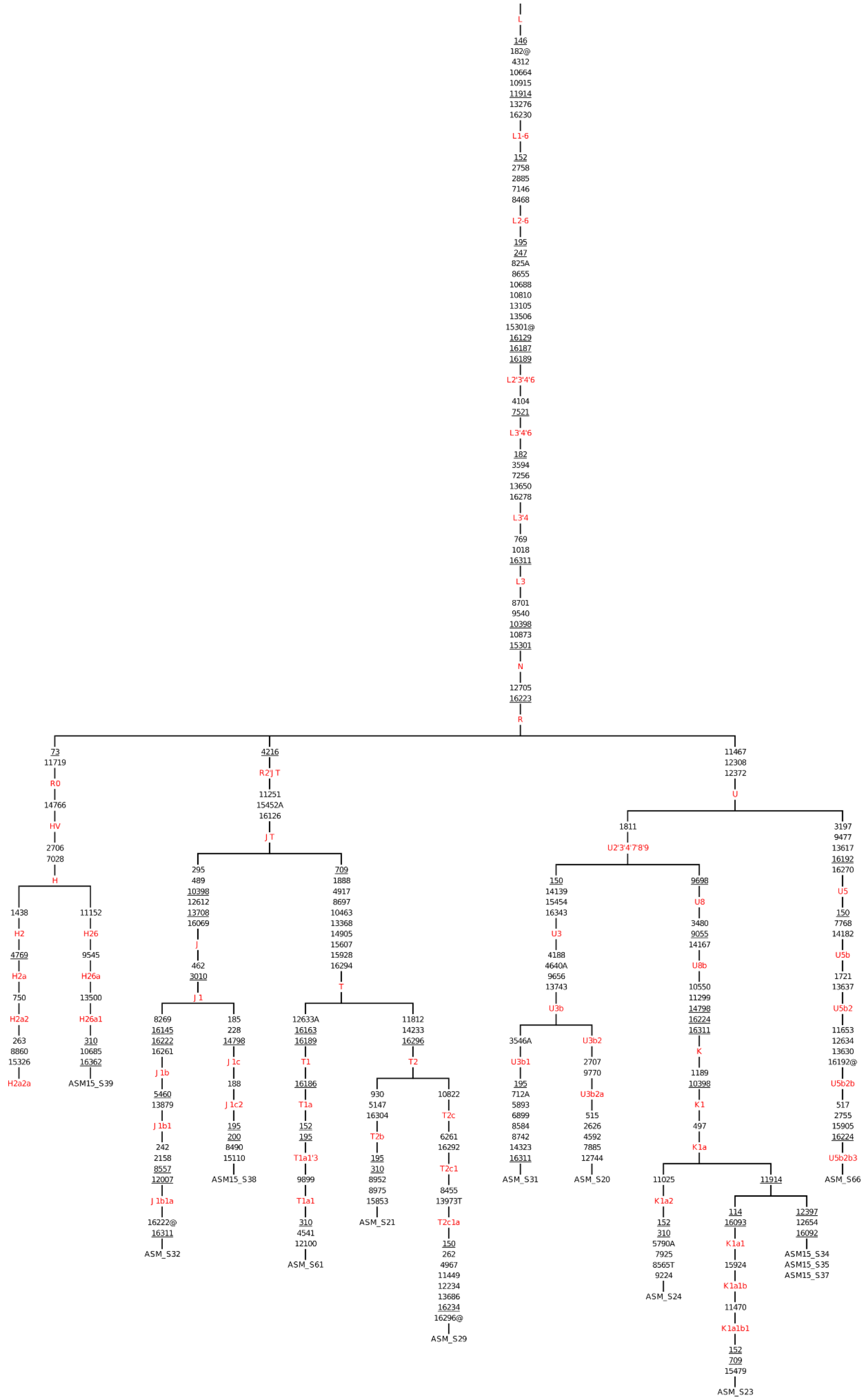

Figure S2. Phylogenetic tree of the 14 complete mitogenomes from the ancient individuals of Amiternum.

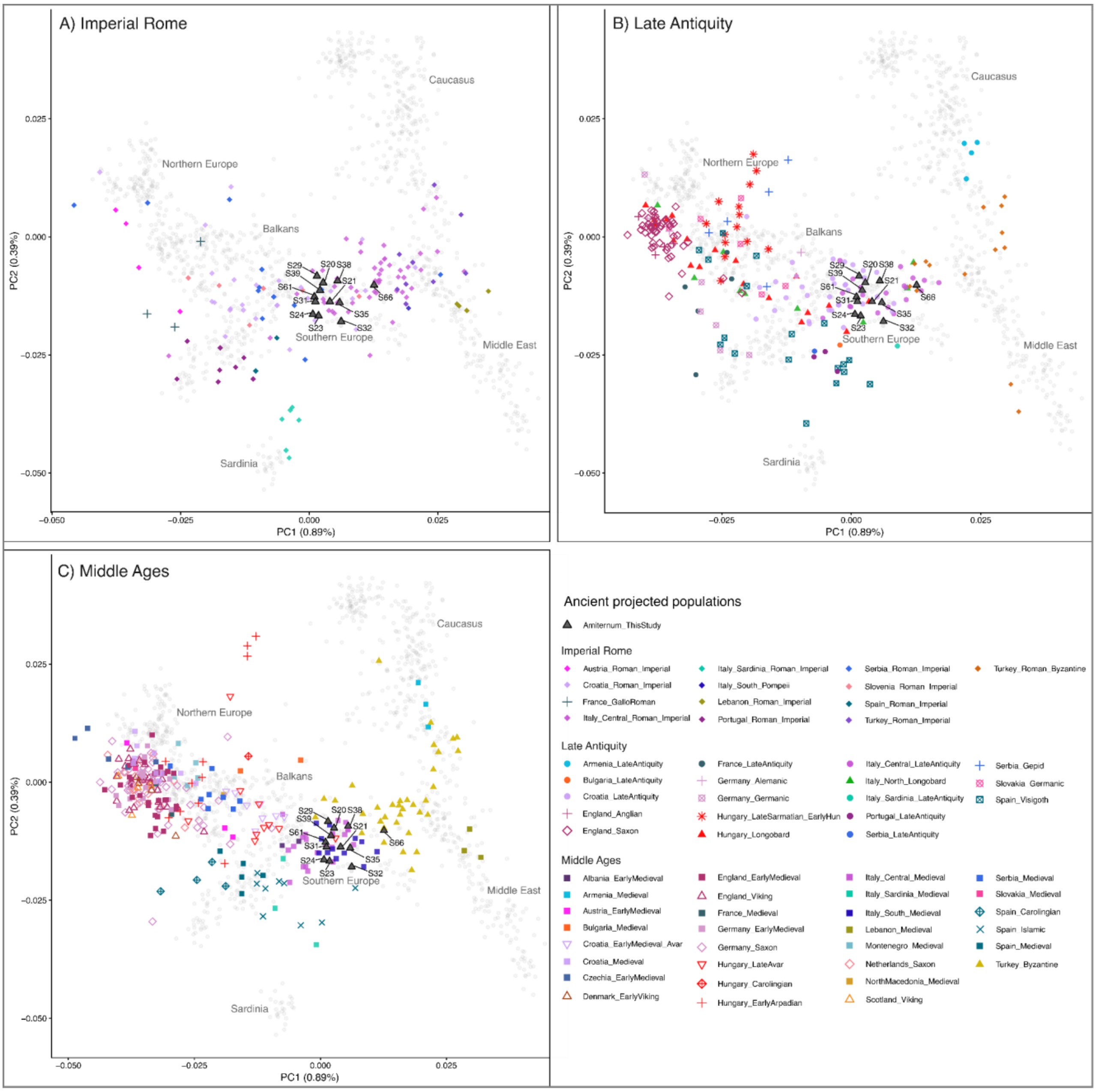

Figure S3. Principal Component Analysis (PCA) of ancient Amiternum individuals in the context of Roman to medieval Europe and Western Asia. PCA plot of 671 published ancient individuals from Europe and Western Asia dated back from Roman to medieval periods (panels A-C, colored symbols) and the 12 ancient individuals from Amiternum (dark grey triangles) projected onto present-day genetic variability (grey dots).

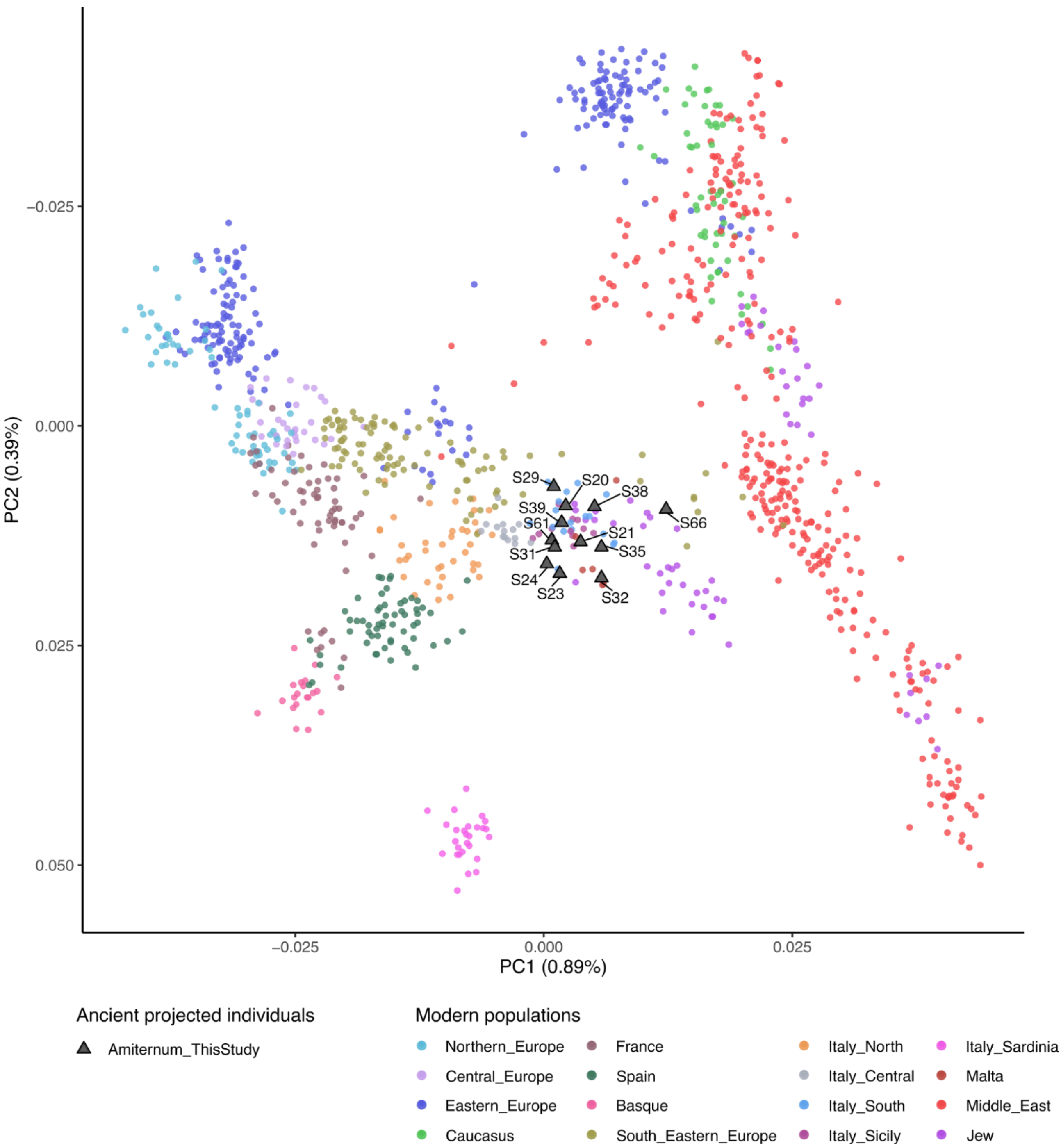

Figure S4. Principal Component Analysis (PCA) of ancient Amiternum individuals in the context of present-day Europe and Western Asia. PCA plot of the 12 ancient individuals from Amiternum (dark grey triangles) projected onto 1124 modern individuals from Europe and Western Asia.

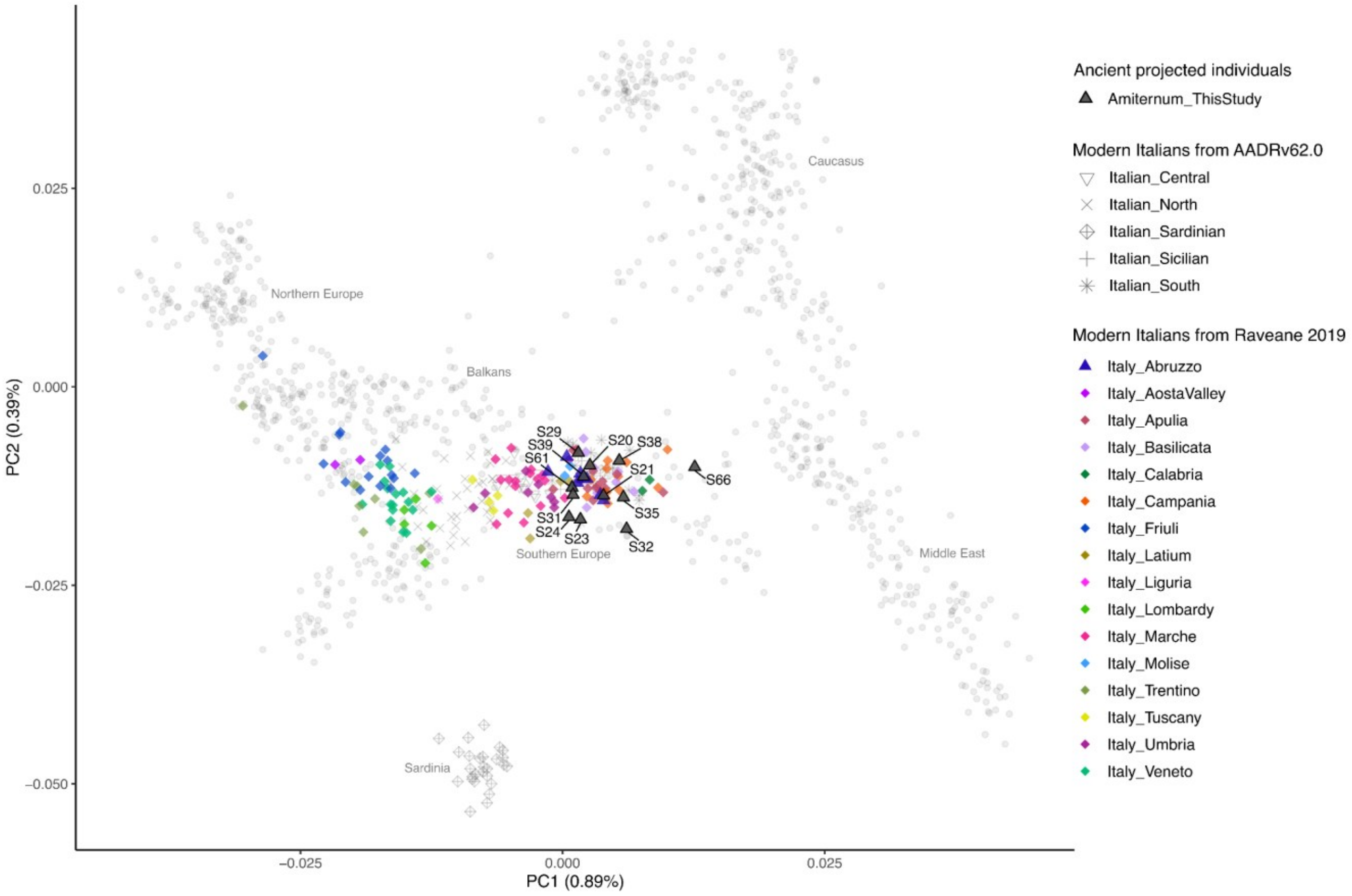

Figure S5. Principal Component Analysis of ancient Amiternum individuals in the context of present-day Italy. Principal Component Analysis (PCA) plot of the 12 ancient individuals from Amiternum (dark grey triangles) and 129 modern Italians from Raveane et al. 2019 (colored symbols) projected onto present-day genetic variability (grey dots). Eight modern individuals from Abruzzo are highlighted by blue triangles.

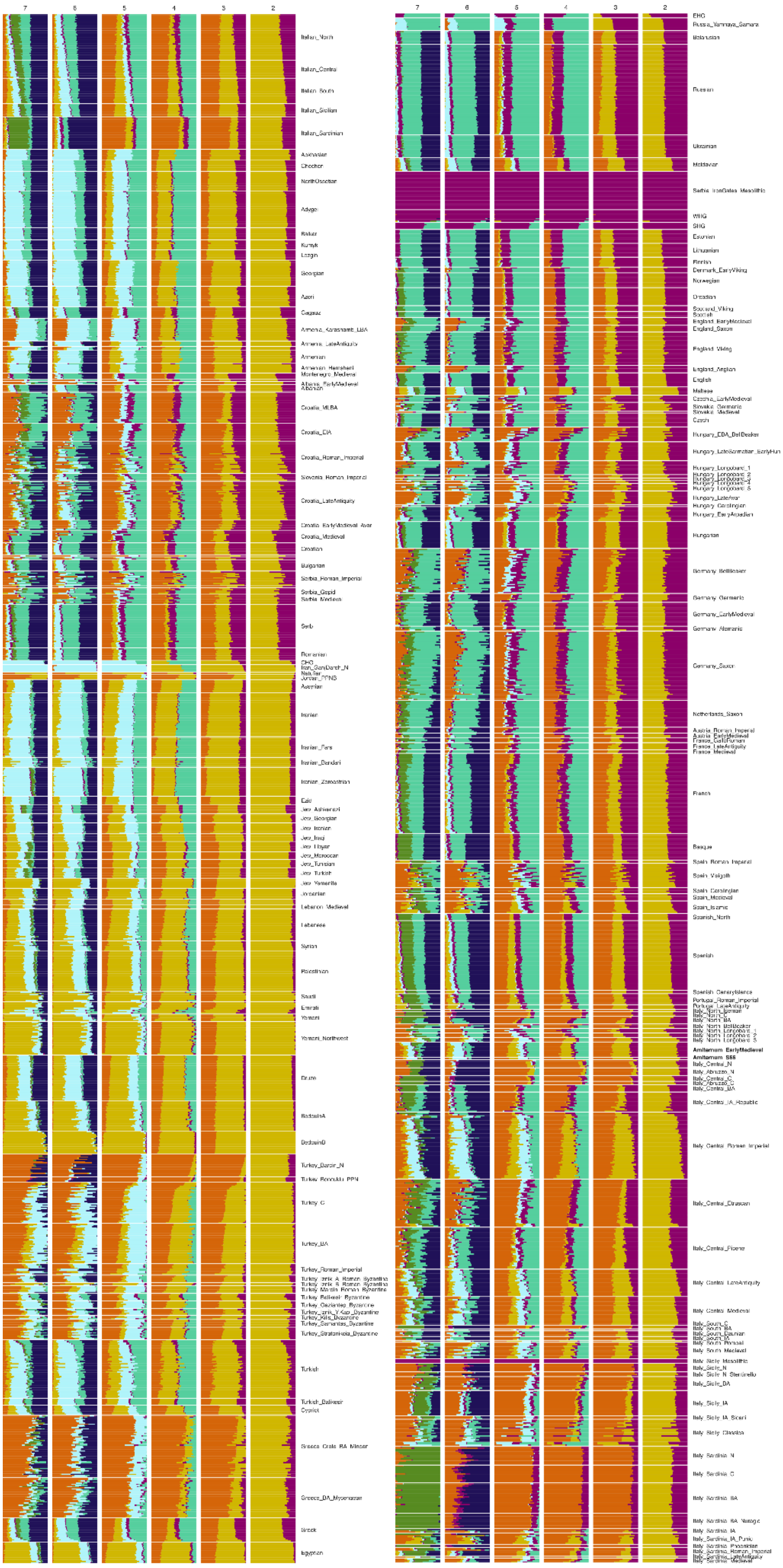

Figure S6. ADMIXTURE analysis of Amiternum individuals among modern and ancient Western Eurasian and North African populations. Unsupervised ADMIXTURE analysis of 1174 modern genomes from Western Eurasia and Northern Africa merged with 1217 published ancient individuals dated back from Epipaleolithic to Middle Ages and the 12 individuals from Amiternum. Here are shown the best runs from K=2 to K=7. Amiternum individuals are indicated in bold.

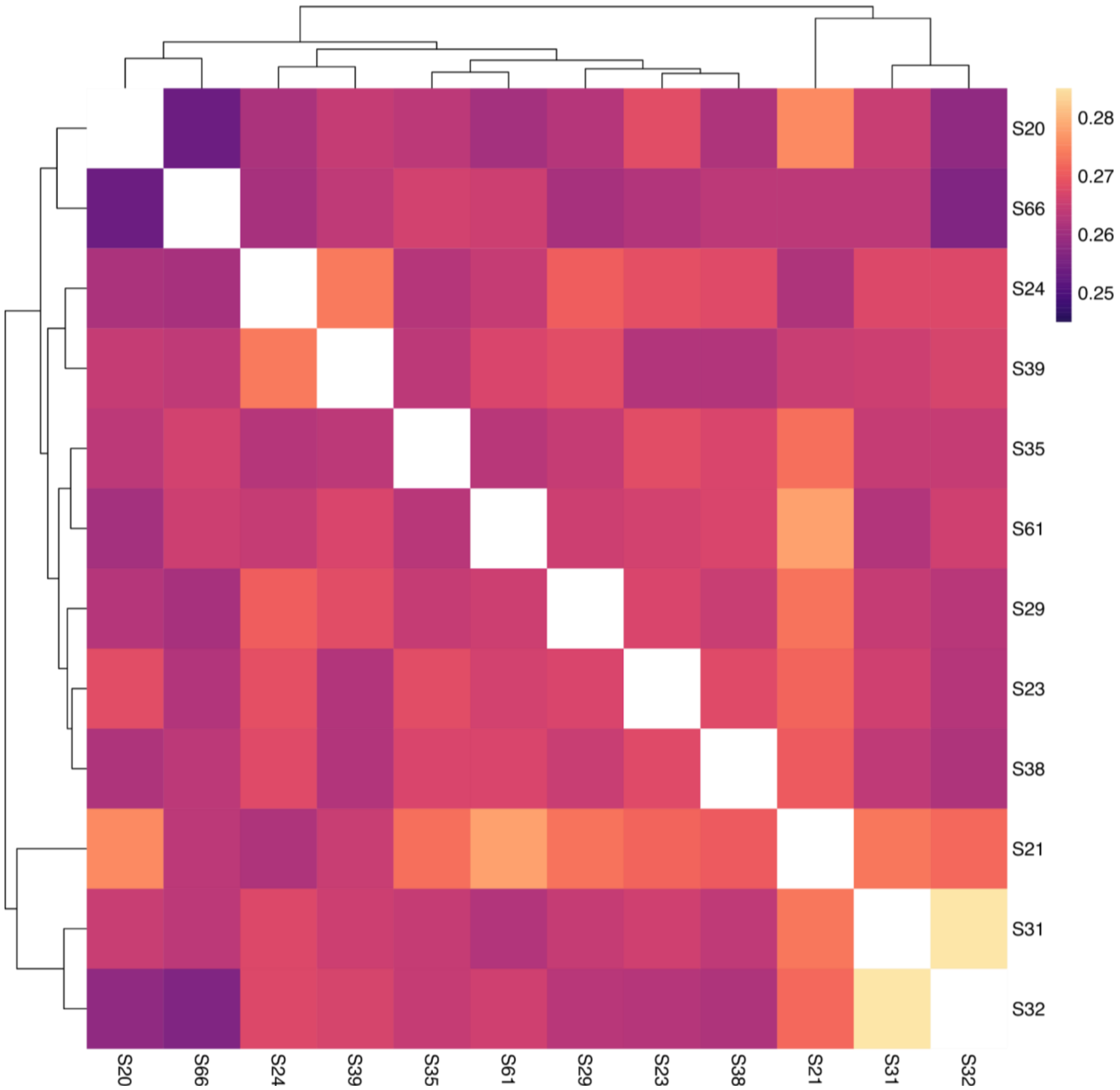

Figure S7. Heatmap showing results of outgroup *f*3 in the form *f*3(Mbuti.DG; X, Y), where X and Y are one of the 12 individuals from Amiternum.

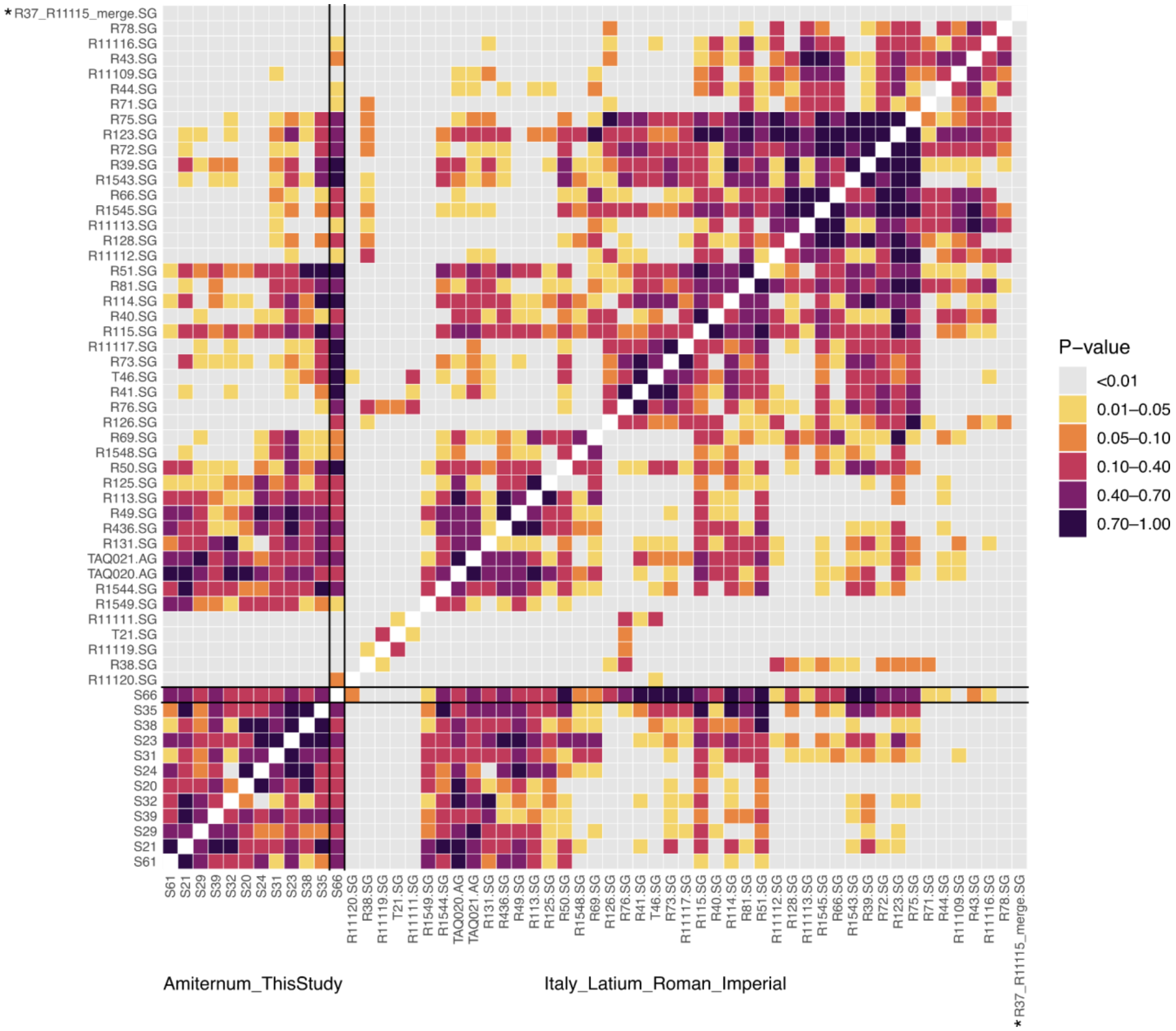

Figure S8. Heatmap of pairwise qpWave tests for 57 individuals (12 Amiternum individuals from this study and 45 published Roman Imperial individuals from Latium). Black lines represent groupings by geographic location and period. Grey-coloured models have a p-value of less than 0.01, and were rejected. Roman individual R37_R11115_merge.SG was identified as an outlier (*) and removed from downstream analyses.

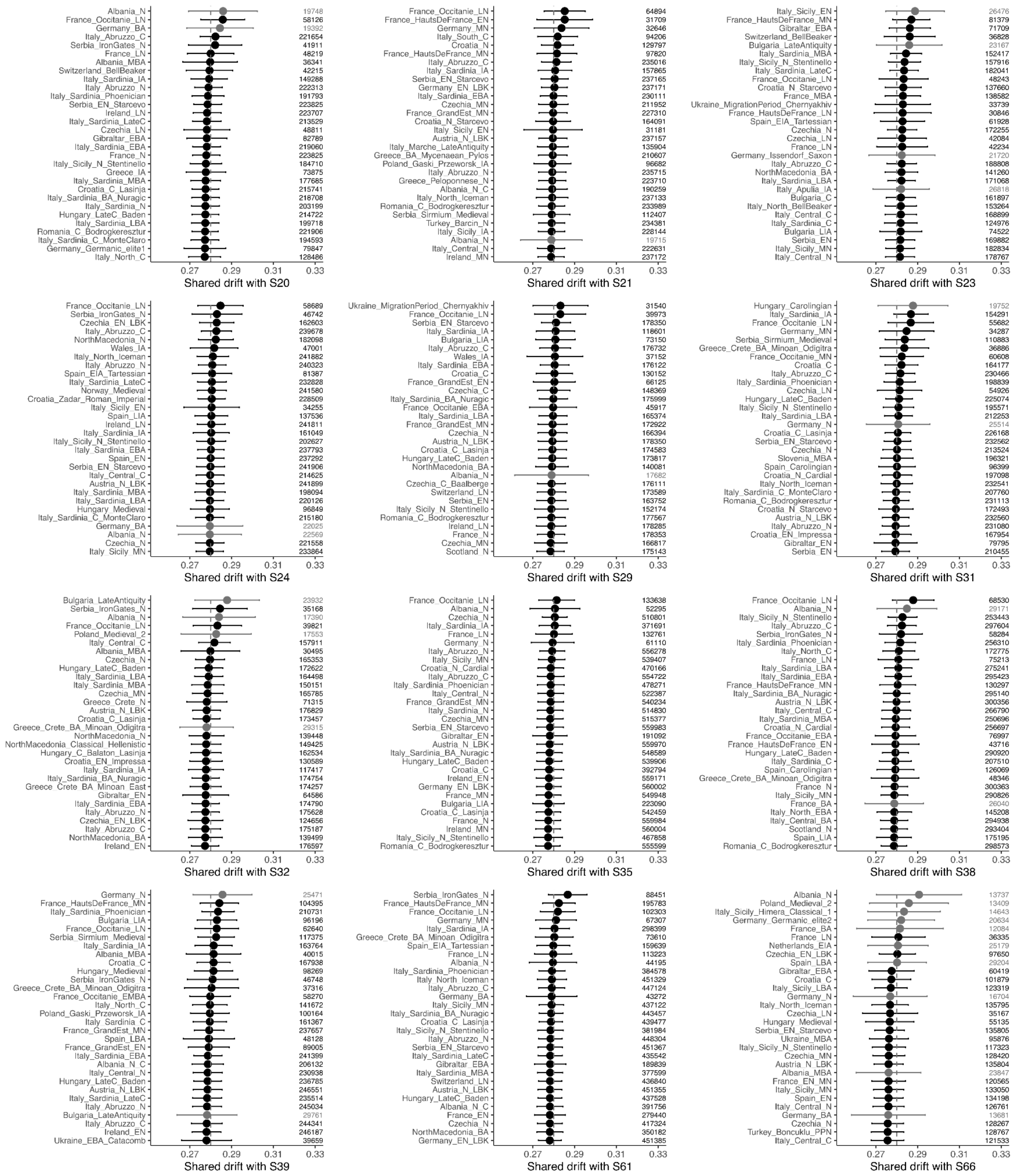

Figure S9. Outgroup *f*3 analysis in the form *f*3(Mbuti.DG; Amiternum, X) measuring shared genetic drift between Amiternum individuals and published ancient populations. Top 30 results of outgroup *f*3 analysis comparing Amiternum individuals with other published ancient populations indicated at the left side of each plot (STAR Methods and Table S10). The number of overlapping SNPs is reported at the right side of each plot and comparisons involving less than 30,000 SNPs are shown in grey.

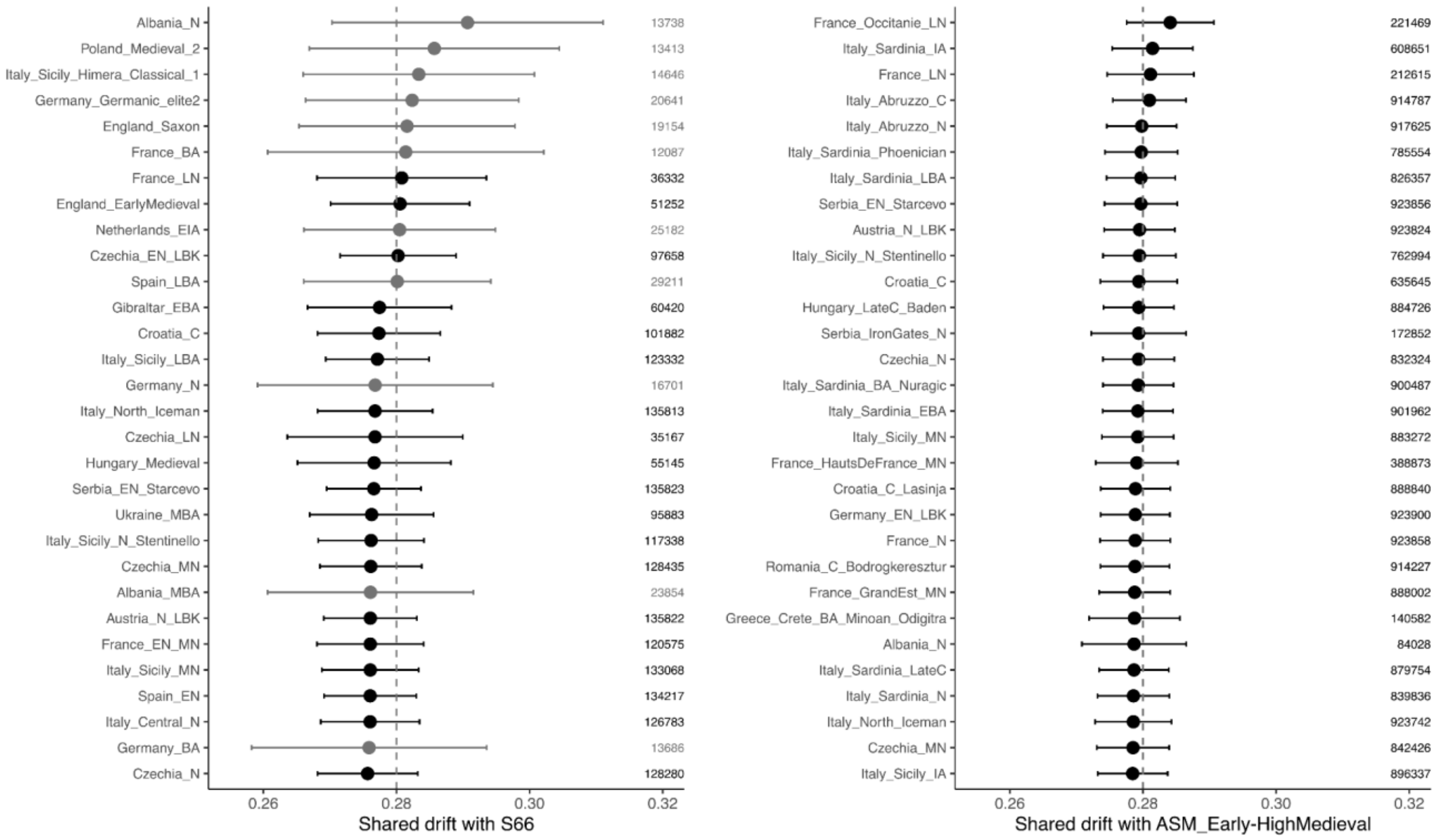

Figure S10. Outgroup *f*3 analysis in the form *f*3(Mbuti.DG; Amiternum, X) measuring shared genetic drift between Amiternum individual S66/ASM_Early-HighMedieval and published ancient populations. Top 30 results of outgroup *f*3 analysis comparing Amiternum individual S66 and ASM_Early-HighMedieval with other published ancient populations indicated at the left side of each plot (STAR Methods and Table S10). The number of overlapping SNPs is reported at the right side of each plot and comparisons involving less than 30,000 SNPs are shown in grey.

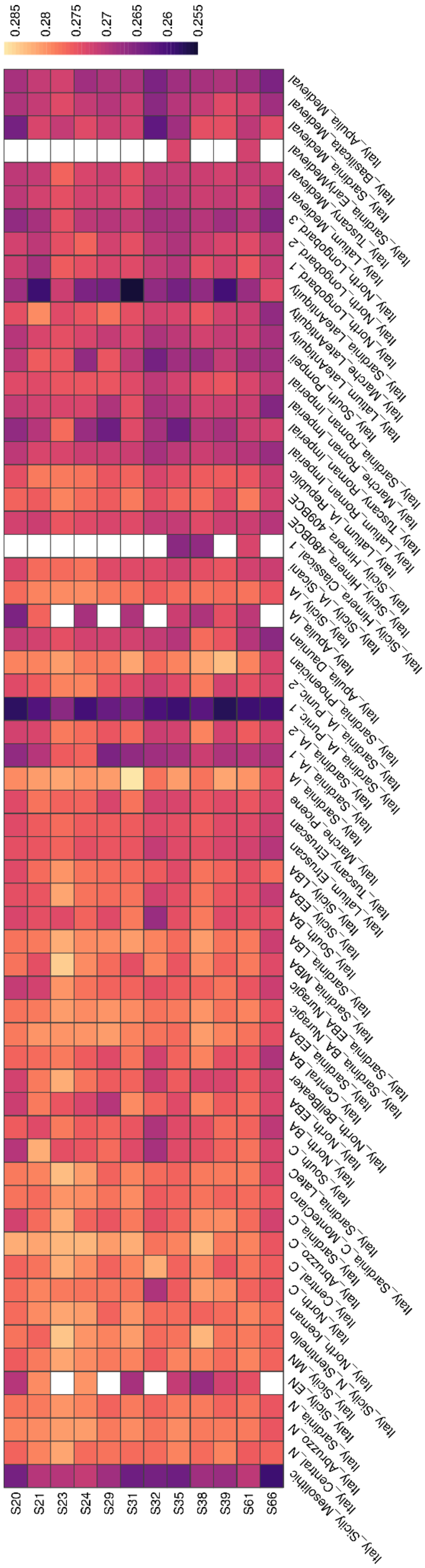

Figure S11. Outgroup *f*3 analysis in the form *f*3(Mbuti.DG; Amiternum, X), where X is an ancient Italian population. Heatmap showing the shared genetic drift between Amiternum individuals and Italian past populations from Mesolithic to Middle Ages (STAR Methods and Table S10). Results with less than 30,000 overlapping SNPs were not represented.

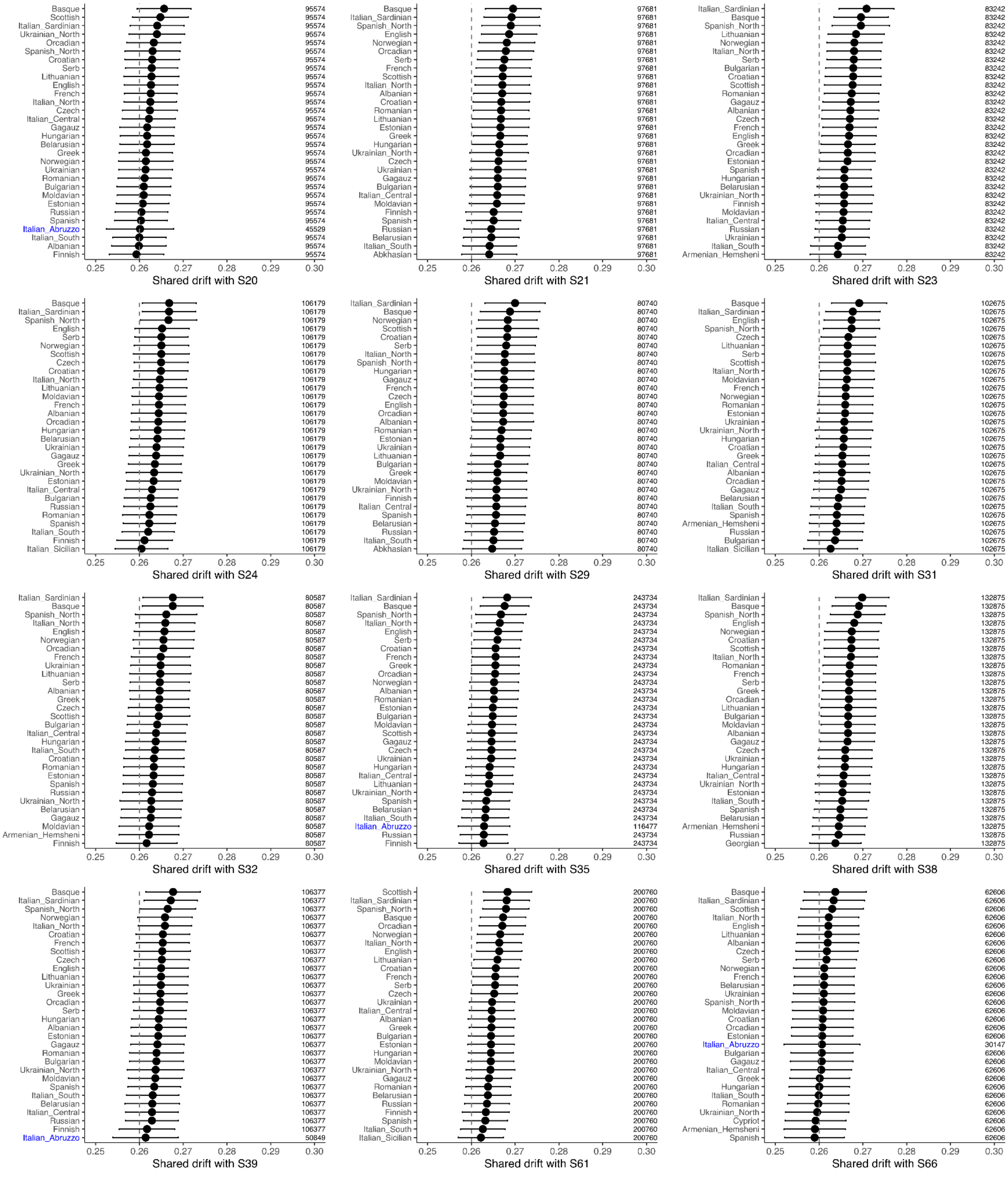

Figure S12. Outgroup *f*3 analysis in the form *f*3(Mbuti.HO; Amiternum, X) measuring shared genetic drift between Amiternum individuals and present-day populations. Top 30 results of outgroup *f*3 analysis comparing Amiternum individuals with published modern populations indicated at the left side of each plot, including eight individuals from Abruzzo highlighted in blue (STAR Methods and Table S12). The number of overlapping SNPs for each comparison is reported at the right side of each plot.

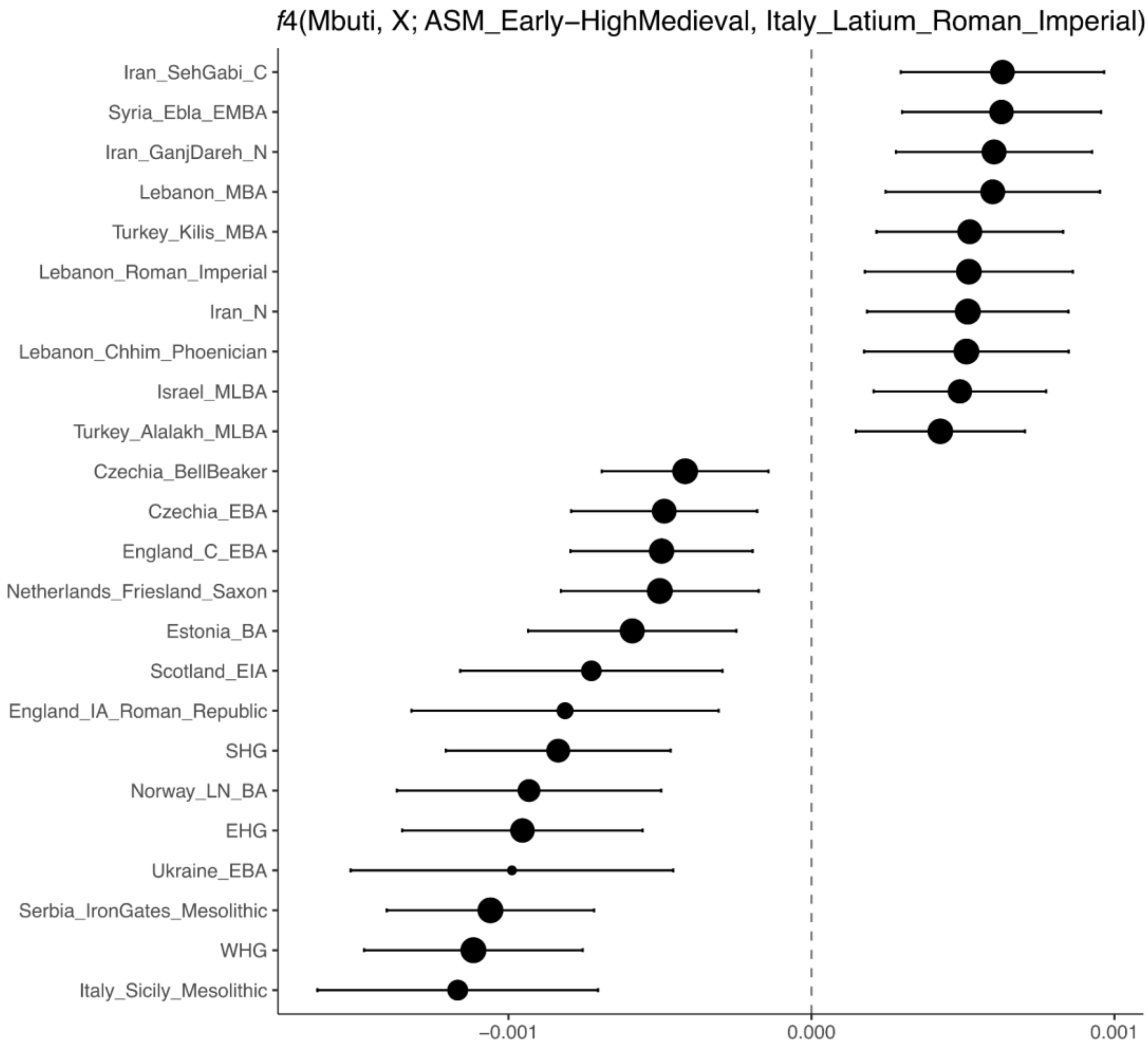

Figure S13. Genetic relationships between ASM_Early-HighMedieval, Italy_Latium_Roman_Imperial and a set of ancient populations in the form *f*4(Mbuti.DG, X; ASM_Early-HighMedieval, Italy_Latium_Roman_Imperial). Here are shown *f*4 values with significant Z-scores (|Z|≥3) and the respective standard errors **(**STAR Methods and Table S13). The point size refers to the number of overlapping SNPs (at least 30,000).

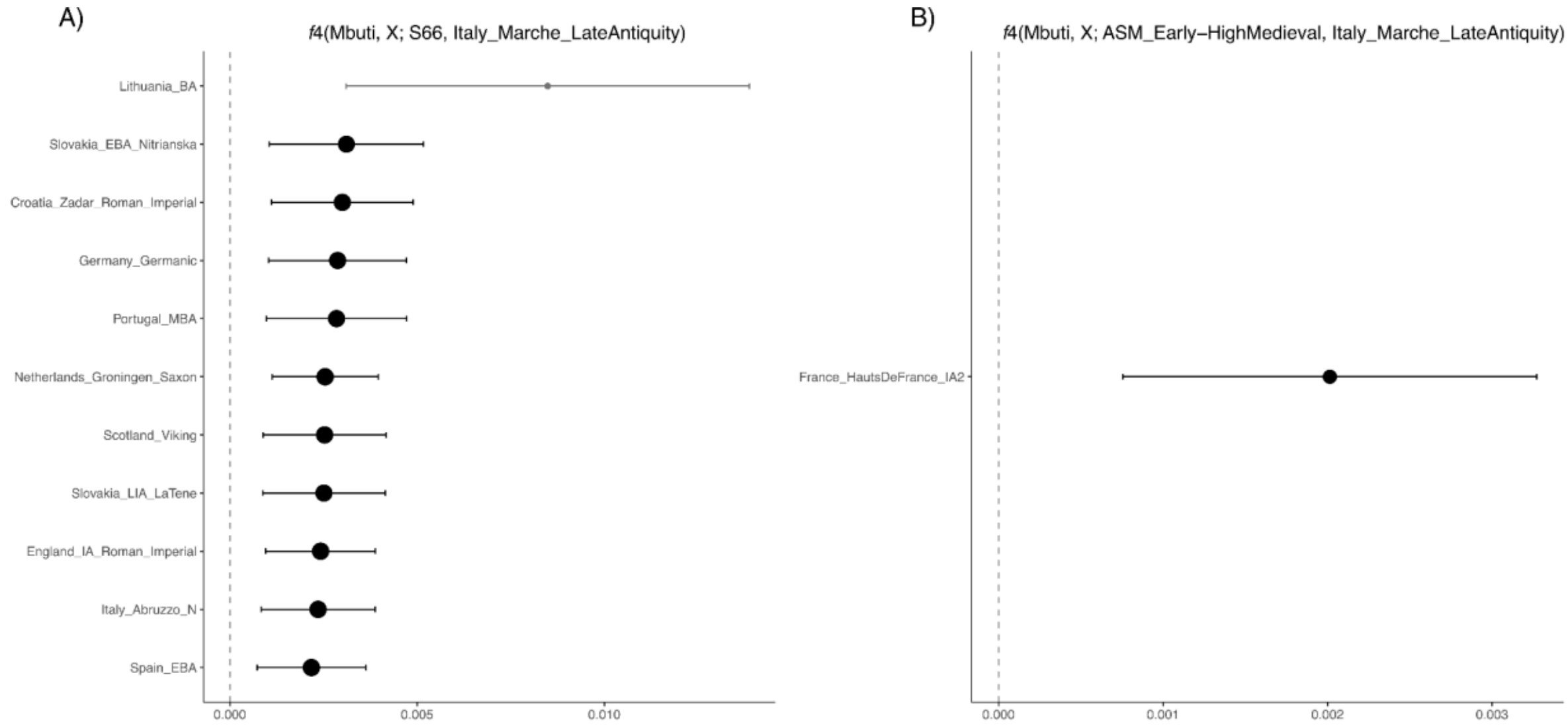

Figure S14. Genetic relationships between ASM_Early-HighMedieval/S66, Italy_Marche_LateAntiquity and a set of ancient populations. A) *f*4 analysis in the form *f*4(Mbuti.DG, X; S66, Italy_Marche_LateAntiquity); B) *f*4 analysis in the form *f*4(Mbuti.DG, X; ASM_Early-HighMedieval, Italy_Marche_LateAntiquity). Here are shown *f*4 values with significant Z-scores (|Z|≥3) and the respective standard errors (STAR Methods and Table S16). The point size refers to the number of overlapping SNPs (at least 30,000).

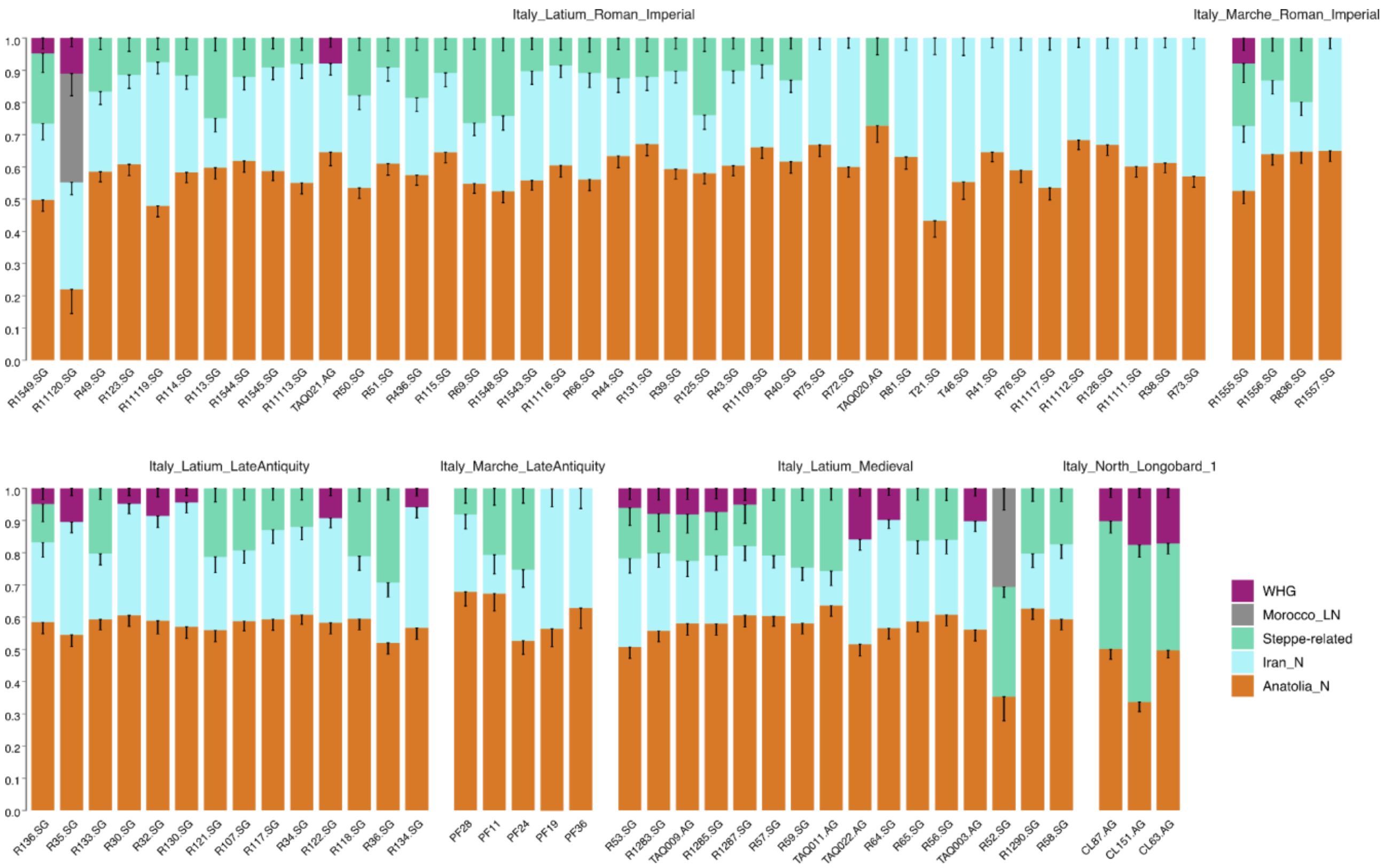

Figure S15. qpAdm-estimated ancestry proportions and respective standard errors for ancient published Italians dated back from Roman Imperial to Medieval time using Western Hunter-Gatherers (WHG), Anatolia Neolithic (Turkey_Barcin_N), Iran Neolithic (Iran_GanjDareh_N), and Steppe-related ancestry (Russia_Yamnaya_Samara) as distal sources. Models shown represent the most parsimonious configurations identified through a model competition framework involving source rotation (STAR Methods and Table S17).

Data S1. Archaeological description of the burials of the Amiternum individuals, related to STAR Methods.

S20

Individual S20 was recovered from Tomb 9, located within the funerary chapel. The burial belongs to the latest funerary phase of the chapel (8th-9th century CE). The grave consisted of a simple earth-cut pit lined with stones and built against wall USM 250 (Figure 2), which formed one side of the structure. The tomb contained a single infant (0-5 years old) deposited in primary position. The individual was laid supine with the upper limbs extended alongside the body and the lower limbs fully extended. Decomposition occurred in a filled environment.

S21

Individual S21 was recovered from Tomb 10, located within the same funerary sector as S20 and dated to the 8th-9th century CE. The burial consisted of a simple earth-cut grave containing a single infant (0–5 years old) in primary deposition. Post-depositional disturbances hindered the preservation of the skeleton, limiting the reconstruction of the original body position; nonetheless, the individual was clearly buried in a supine position.

S23-S24

Individual S23 was recovered from Tomb 11, a repeatedly reused burial structure containing two primary inhumations (S23 and S24). The grave, dated to the 10th–11th century CE, was located outside the apse of the Lombard cathedral, between walls USM 250 and 382 (Figure 2). It consisted of an earth-cut pit lined at the head and foot with reused stone slabs. The head slab consisted of a reused fragment of a Roman funerary inscription, which projected beyond the grave and probably functioned as a grave marker. S23, an infant aged 3-4 years, was deposited supine with extended limbs. The burial represents the latest funerary event within the tomb.

Individual S24 represents an earlier burial within Tomb 11. The infant (0-2 years old) was deposited supine with the head turned to the left and the arms slightly flexed, resting on the pelvis. The burial was partially disturbed by the later deposition of S23. The reuse of the same funerary structure demonstrates a prolonged commemorative use of the tomb during the 10th–11th century.

S28-S29

Individual S29 was recovered from Tomb 14, a double burial dated to the 8th–9th century CE and located outside the apse of the Lombard cathedral. The grave was constructed using limestone blocks, roof tiles placed as a cover, and a reused brick fragment as the base. S29, an infant aged around 1 year, was found together with another infant, named S28. Individual S29 was found lying supine with extended limbs, whereas S28 was represented only by a small number of skeletal elements. Decomposition occurred in an empty space, suggesting the presence of a void within the burial structure.

S31

Individual S31 was recovered from Tomb 16, located alongside wall USM 257 (Figure 2) and belonging to the 8th–9th century funerary phase. The grave consisted of a stone-lined earth-cut pit containing a single infant (2-3 years old) in primary deposition. The individual was laid supine with extended limbs. The forward displacement of the skull suggests that the head may originally have rested on a perishable support.

S32

Individual S32 was recovered from Tomb 17, located approximately 50 cm from Tomb 16 and built against wall USM 257 (Figure 2). The burial, dated to the 9th century CE, consisted of a stone-lined earth-cut grave, likely originally covered by a wooden structure. The individual, an infant aged around 5 years, was deposited supine with the arms flexed over the abdomen and the ankles crossed. Decomposition occurred in an empty space.

S34-S35

Individuals S34 and S35 are the two infants (aged 3-4 years and 5-6 years, respectively) recovered from Tomb 18, a double burial dated to the 8th–9th century CE. The grave consisted of an earth-cut pit marked by two large amphora fragments, one placed near the heads and another covering the thoracic area. Both infants were deposited simultaneously in primary position. The two bodies were placed with their heads side by side, and S34 partially overlapped S35, likely because of the limited dimensions of the grave.

S37-S38

Individual S37, an infant aged 0-5 years, was recovered from Tomb 19, located close to Tomb 18 and dated to the 8th–9th century CE. The burial consisted of an earth-cut grave covered by a roof tile leaning against wall USM 256 (Figure 2). The tomb contained S37 in primary deposition and S38 in reduction. The grave yielded the only significant funerary offering associated with the individuals analyzed in this study, namely a plain two-handled ceramic vessel. The skeleton of S38, the reduced burial within Tomb 19, belonged to a juvenile individual and was found in secondary position.

S39

Individual S39 was recovered from Tomb 21, located alongside wall USM 250 (Figure 2) and dated to the 9th century CE. The grave consisted of a simple earth-cut pit partially disturbed by the later construction of Tomb 11. The individual, an infant aged 1.5 years, was deposited singly in primary position, lying supine with extended limbs. Decomposition occurred in an empty space.

S58

Individual S58 was recovered from Tomb 37. The burial, dated to the 9-10th century CE, was built against wall USM 918 (Figure 2), belonging to the lateral nave of the Lombard cathedral. The grave consisted of a stone-lined earth-cut pit covered by a large slab. Osteological analysis identified the individual as a young adult female (17–25 years old). The body was deposited supine with arms crossed over the abdomen.

S61

Individual S61 was recovered from Tomb 40, located between walls USM 1177 and 280 (Figure 2). The burial, dated to the 8th-9th century CE, was heavily disturbed by later construction activities associated with the closure of a side entrance to the cathedral. Despite the poor preservation of the remains, the available skeletal elements indicate that the infant, aged 0-5 years, had originally been deposited in a supine position.

S66

Individual S66 was recovered from a stone-lined grave between walls USM 305, 943 and 1503 (Figure 2). The burial consisted of an earth-cut grave with side walls built in dry stone and likely covered by a perishable superstructure. The adult male (35–50 years old) was deposited singly in primary position, lying supine with extended limbs. Radiocarbon dating places the burial between the 3rd-4th century CE, making S66 the earliest individual included in the dataset and the only burial predating the medieval funerary phase associated with the cathedral.
